# When Development Falls out of Sync: A Developmental Timing Framework for Learning Disabilities

**DOI:** 10.3390/brainsci16090987

**Published:** 2026-09-17

**Authors:** Gerry Leisman

**Affiliations:** Movement and Cognition Laboratory, Department of Physical Therapy, University of Haifa, Haifa 3498838, Israel; g.leisman@edu.haifa.ac.il

**Keywords:** developmental timing, maturational asynchrony, learning disabilities, developmental systems, neuroeducation, dyslexia, ADHD, dyscalculia, developmental neuroscience, school readiness, developmental cascades, longitudinal development

## Abstract

**Highlights:**

**What are the main findings?**

- Proposes a developmental timing framework in which learning disabilities arise from maturational asynchrony among interacting neurocognitive systems.
- Integrates evidence from developmental neuroscience, cognitive psychology, and education to explain heterogeneity, comorbidity, and developmental cascades across dyslexia, ADHD, dyscalculia, and mixed learning profiles.

**What are the implications of the main findings?**

- Generates testable predictions regarding developmental trajectories, longitudinal assessment, and timing-sensitive educational intervention.
- Supports developmentally informed assessment and instruction by emphasizing developmental readiness and cross-system coordination alongside conventional diagnostic classifications.

**Abstract:**

Learning disabilities are traditionally conceptualized as domain-specific disorders arising from deficits in reading, attention, or numerical processing. Although these approaches have substantially advanced understanding of dyslexia, attention-deficit/hyperactivity disorder (ADHD), and dyscalculia, they do not fully explain the marked heterogeneity, developmental variability, frequent comorbidity, or changing learning trajectories observed across children. This conceptual review proposes a developmental timing framework in which learning disabilities emerge, in many cases, from maturational asynchrony among interacting neural, cognitive, linguistic, sensorimotor, and executive systems rather than from isolated deficits within a single domain. Drawing on evidence from developmental neuroscience, cognitive psychology, neuroeducation, developmental systems theory, and learning sciences, we synthesize research on the developmental coordination of language, executive function, attention, motor control, working memory, symbolic learning, and numerical cognition across childhood. Within this framework, dyslexia, ADHD-related learning difficulties, dyscalculia, and mixed learning profiles are interpreted as distinct developmental expressions of differing patterns of cross-system asynchrony interacting with the increasing cognitive demands of formal education. We distinguish maturational asynchrony from related concepts, including developmental variability, developmental cascades, and multiple-deficit models, and propose measurable indicators through which the framework may be empirically evaluated. The framework further generates testable predictions concerning longitudinal developmental trajectories, educational transitions, developmental fit, and timing-sensitive intervention. Rather than replacing established cognitive accounts of learning disabilities, the developmental timing framework provides an integrative developmental architecture within which existing theories can be understood while offering a basis for future longitudinal and intervention research. This perspective highlights the importance of aligning educational expectations and instructional practices with children’s evolving neurodevelopmental readiness rather than chronological age alone.

## 1. Introduction: Why Learning Disabilities Require a Developmental Timing Framework

Understanding how children learn has become a central objective of developmental neuroscience and neuroeducation. Advances in cognitive neuroscience have substantially improved our understanding of the neural systems underlying language, attention, executive function, memory, motor control, and numerical cognition, while educational research has identified instructional approaches that facilitate learning across diverse developmental profiles. Yet an important question remains comparatively underexplored: how do differences in the timing and coordination of neurodevelopment influence children’s readiness to acquire academic skills? Although considerable progress has been made in identifying the neural and cognitive mechanisms underlying specific learning disabilities, comparatively less attention has been devoted to understanding how interactions among developing systems unfold across time, how their functional coordination changes during development, and how variation in these developmental trajectories contributes to the emergence of learning difficulties.

Although considerable progress has been made in identifying the neural and cognitive mechanisms underlying specific learning disabilities, less attention has been devoted to understanding how interactions among developing systems unfold across time and how variation in their maturation contributes to the emergence of learning difficulties. Addressing this question is fundamental to neuroeducation because successful learning depends not only on the integrity of individual cognitive systems but also on the developmental coordination of multiple neural processes that must become functionally integrated as children encounter increasingly complex educational demands.

Here, we refer to developmental timing as the rate, sequence, and functional coordination with which interacting neurocognitive systems become sufficiently integrated to support emerging academic skills. It therefore differs from chronological age, generalized developmental delay, or developmental variability alone. Two children of the same age may demonstrate similar levels of performance on individual component skills yet differ substantially in the degree to which those skills have become coordinated for efficient academic learning. Throughout this review, maturational asynchrony refers to differences in the developmental timing of interacting systems that result in temporary or persistent mismatches between developmental organization and educational demands.

Learning disabilities are traditionally classified according to the academic domain in which impairment becomes most apparent. Dyslexia is primarily characterized by persistent difficulties in accurate or fluent word recognition, decoding, and spelling. Attention-deficit/hyperactivity disorder (ADHD), including predominantly inattentive presentations historically described as attention deficit disorder (ADD), is characterized by impairments in attention regulation, inhibitory control, activity regulation, and executive functioning. Dyscalculia is defined principally by persistent difficulties in number processing, arithmetic fluency, calculation, and mathematical reasoning. These classifications have substantial clinical value because they guide diagnosis, educational planning, intervention, and communication among clinicians, educators, researchers, and families. However, they also risk implying that learning disabilities reflect isolated impairments confined to specific academic domains. From a developmental perspective, this interpretation is incomplete. Reading, attention, and mathematical reasoning are not independent cognitive functions but complex developmental achievements that emerge through the progressive coordination of multiple neurocognitive systems maturing at different rates and becoming increasingly integrated throughout childhood [1,2,3,4].

The need for a developmental perspective becomes particularly evident when considering that the academic abilities used to define learning disabilities are themselves culturally acquired rather than biologically primary functions. Reading requires the coordinated development of visual perception, spoken language, phonological processing, orthographic learning, vocabulary, eye-movement control, selective attention, working memory, and fluent integration of these component processes. Mathematics similarly depends upon the progressive integration of approximate magnitude representations, symbolic number knowledge, exact quantity processing, spatial cognition, procedural sequencing, working memory, executive control, and abstract reasoning. Likewise, successful classroom attention and self-regulation require coordinated interactions among arousal regulation, motor inhibition, sustained attention, motivational processes, executive functioning, reward sensitivity, and future-oriented behavioral control. In every instance, the observable academic skill represents the culmination of a prolonged developmental process during which multiple neural and cognitive systems must mature sufficiently and become coordinated with one another to support increasingly complex forms of learning [5,6,7,8,9].

Although several developmental frameworks emphasize dynamic interactions among cognitive systems, comparatively few explicitly focus on how variation in developmental timing and cross-system coordination influences children’s readiness for formal learning. Nevertheless, relatively few theoretical frameworks explicitly address how differences in developmental timing influence children’s capacity to benefit from educational instruction. Educational success depends not only on whether particular cognitive abilities eventually develop, but also on whether interacting systems become sufficiently coordinated at the developmental stages during which formal instruction requires their simultaneous engagement. Consequently, understanding developmental timing provides an essential bridge between neuroscience and educational practice by explaining why children with comparable intellectual ability frequently demonstrate markedly different patterns of learning, academic achievement, and responsiveness to instruction.

We propose a developmental timing framework associated with learning disabilities in which academic achievement depends not only upon the acquisition of individual component skills but also upon the temporal organization of their development. Successful learning requires multiple neurocognitive systems to emerge in appropriate developmental sequences, mature at compatible rates, and become functionally synchronized as educational demands progressively increase. Children may possess many of the component capacities necessary for successful learning, yet if these systems mature unevenly or become coordinated only after formal instruction requires their integration, learning may become inefficient, unstable, or vulnerable to disruption. Developmental timing represents more than chronological maturation; it reflects the evolving coordination among interacting biological, cognitive, motor, and environmental systems that collectively support adaptive learning. This perspective is consistent with developmental systems theory, which conceptualizes cognitive outcomes as emerging from continuous interactions among biological maturation, behavior, experience, and environmental context rather than from isolated causal mechanisms [10,11,12].

The framework does not seek to replace established cognitive theories of learning disabilities. Rather, it is intended as a higher-level developmental account that organizes existing mechanisms within a common temporal architecture. Its central proposition is that the educational consequences of cognitive strengths and weaknesses depend not only on their presence but also on the developmental timing with which interacting systems become coordinated. This proposition generates empirically testable predictions regarding longitudinal developmental trajectories, developmental fit, cross-system coordination, and responsiveness to intervention, which are considered later in the review.

A developmental timing perspective complements rather than replaces established domain-specific theories of learning disabilities. Contemporary models of dyslexia have demonstrated the importance of phonological processing, orthographic learning, language development, and cross-linguistic differences in orthographic transparency [13,14,15]. Similarly, executive dysfunction, motivational regulation, reward processing, delay-related mechanisms, temporal processing, and state regulation remain central to current theoretical accounts of ADHD [16,17,18,19,20]. Research on dyscalculia has likewise identified the importance of numerical magnitude representations, symbolic number processing, arithmetic fluency, spatial cognition, and individual variability in mathematical development [21,22,23,24]. Collectively, these models have substantially advanced our understanding of disorder-specific mechanisms. However, they primarily focus on the cognitive processes most closely associated with observable academic impairment. A developmental timing framework proposed here asks a complementary question: how do the multiple neural systems supporting reading, attention, executive control, and mathematical reasoning become coordinated across development, and what are the developmental consequences when this coordination is delayed, developmentally asynchronous, or insufficiently integrated relative to educational demands?

## 2. Developmental Timing, Maturational Asynchrony, and Developmental Cascades

A developmental timing framework begins from the premise that cognitive development is not the simple accumulation of independent abilities but the progressive organization of interacting biological, neural, cognitive, motor, linguistic, emotional, and environmental systems across time. Academic competence is proposed to emerge as these partially independent systems mature, interact, and become sufficiently coordinated to support increasingly complex forms of learning. Reading, writing, mathematical reasoning, sustained attention, executive control, and self-regulated learning are not isolated cognitive achievements but emergent properties of developmental coordination. Their successful acquisition depends not only on the maturation of individual component systems but also on the temporal alignment of those systems with one another and with the educational experiences that promote their integration [25,26,27].

This perspective is grounded in developmental systems theory, which views development as the product of continuous reciprocal interactions among biological maturation, neural plasticity, behavior, experience, and environmental context rather than the expression of isolated causal mechanisms [10,11,12]. Within this framework, the developmental significance of any emerging ability depends not only on its intrinsic characteristics but also on the developmental state of the systems with which it must ultimately interact. Skills acquired early can provide the foundation for increasingly complex forms of learning, whereas delayed or unstable development may restrict subsequent opportunities for practice, refinement, and integration. Development reflects both the acquisition of individual abilities and the evolving organization of relationships among those abilities as children encounter progressively greater cognitive, social, and educational demands.

An important implication of this perspective is that development is constrained by sequential dependencies among interacting systems. Later-emerging competencies do not arise independently but build upon the successful organization of earlier neural and cognitive processes. Language development supports phonological awareness; phonological awareness facilitates literacy acquisition; early numerical representations support symbolic mathematics; executive functions increasingly coordinate goal-directed learning; and perceptual, motor, attentional, and emotional systems progressively contribute to adaptive classroom behavior. Consequently, variation in the developmental trajectory of one component system can influence the organization of many others, even when each system reaches an apparently typical level of maturity. The educational consequences of developmental variation depend not simply on whether individual abilities emerge but on whether they become available in an appropriate sequence and within a developmental context that permits their functional integration.

The present framework is organized around four interrelated concepts that together describe how neurodevelopmental variation may influence learning outcomes. Developmental timing refers to the rate, sequence, stability, and functional coordination with which interacting neurocognitive systems mature and become available for increasingly complex learning. Operationally, it can be examined by longitudinal measures of developmental trajectories, cross-system synchrony, and the timing with which developing systems become sufficiently coordinated to support emerging academic skills [25]. Candidate indicators could include age-referenced growth slopes, within-child differences among developmental trajectories, cross-lagged relations among component skills, changes in cross-domain covariance over time, and the temporal relation between such changes and the onset of specific educational demands. Maturational asynchrony refers to measurable differences in the developmental tempo of interacting systems that normally function together. It is operationally expressed as divergence in developmental trajectories or delayed functional coordination among systems relative to the educational demands placed upon the child [28]. Asynchrony could therefore be quantified as the magnitude and persistence of within-child divergence among standardized developmental trajectories of functionally interacting systems.

Developmental cascades describe the process by which relatively small early differences in developmental timing alter later opportunities for practice, feedback, motivation, self-regulation, and skill acquisition, thereby progressively reshaping developmental trajectories [29]. Operationally, developmental cascades are reflected by longitudinal changes in which early developmental differences predict increasingly broader academic, behavioral, or psychosocial outcomes over time. Finally, school-readiness mismatch refers to the discrepancy that arises when educational expectations are determined largely by chronological age while neurocognitive readiness varies substantially across children because interacting developmental systems have reached different stages of functional organization [30]. Mismatch can be evaluated by comparing the developmental organization of relevant neurocognitive systems with the timing and complexity of educational demands. These concepts provide a developmental framework for understanding learning disabilities as dynamic outcomes emerging from interactions among biological maturation, experience-dependent plasticity, and the educational environment rather than as isolated impairments confined to individual academic domains.

Although each of these concepts has appeared previously within the developmental systems, developmental cascade, or educational readiness literature, the present framework integrates them into a single explanatory model centered on developmental timing. The novel proposition is that the developmental coordination of interacting systems, rather than the maturity of individual systems alone, constitutes a distinct and potentially measurable source of variability in learning outcomes. The framework focuses on cross-system synchronization as a developmental mechanism linking neurodevelopment to educational performance.

### 2.1. Developmental Timing

Developmental timing refers to the temporal organization of maturation across interacting neurocognitive systems. It encompasses not only when particular functions first emerge, but also the rate at which they stabilize, the sequence in which they develop, the extent to which they become coordinated with other systems, and whether they are sufficiently organized when environmental and educational demands require their integrated operation. Development is concerned not simply with the eventual acquisition of phonological awareness, inhibitory control, working memory, number sense, or symbolic reasoning but with the developmental processes through which these capacities become sufficiently integrated to support increasingly complex forms of learning [25].

Development is inherently sequential because later-emerging competencies depend on the successful organization of earlier systems. Neural maturation proceeds through a series of developmental dependencies in which the acquisition of one function creates the conditions necessary for the emergence of subsequent abilities. Consequently, developmental timing reflects more than chronological age; it reflects the progressive construction of functional networks that permit increasingly sophisticated cognitive, behavioral, and academic performance. Individual differences in the timing of these developmental processes can influence not only when particular skills emerge but also how efficiently they are integrated into broader neurocognitive systems.

This principle is evident across multiple domains of learning. In literacy, early speech perception contributes to the establishment of stable phonological representations [31]. These representations provide the foundation for grapheme–phoneme mapping [32], which in turn supports orthographic learning [33] and the gradual development of fluent word recognition [34,35]. Likewise, mathematical development proceeds through a series of increasingly integrated representations. Early non-symbolic quantity processing supports the acquisition of number words [36,37], which facilitates symbolic number mapping [37], arithmetic procedures [38], and progressively more abstract mathematical reasoning [38]. Comparable developmental dependencies characterize attention and self-regulation, where arousal regulation, motor inhibition, delay tolerance, working memory, executive control, and goal-directed behavior become increasingly coordinated as children adapt to structured educational environments.

Developmental timing should not be interpreted as a rigid maturational timetable. Although sensitive periods and developmental windows are well established, they do not imply that learning is restricted to narrowly defined ages. Rather, they indicate that the effectiveness of experience depends upon the developmental state of the underlying neural systems at the time the experience occurs [39,40,41]. The same educational experience may produce markedly different outcomes depending on whether the relevant neurocognitive systems are sufficiently organized to utilize it. Print exposure, for example, is likely to facilitate reading acquisition when phonological, linguistic, and attentional systems are adequately coordinated but may yield limited benefit or considerable frustration when these systems remain unstable. Similarly, arithmetic instruction is most effective when quantity representations, symbolic number knowledge, language, spatial processing, and working memory have achieved sufficient functional integration to support meaningful learning.

This distinction has important implications for understanding learning disabilities. Many academic skills depend on the coordinated operation of multiple component processes that mature over extended developmental periods [42]. Successful reading requires the integration of auditory discrimination, phonological awareness, oral language, visual-symbol recognition, attention, oculomotor control, and working memory into a rapidly functioning decoding system [43]. Mathematical competence depends upon the coordination of non-symbolic magnitude representations, number words, Arabic numerals, spatial representations, procedural learning, executive sequencing, and working memory [36,44]. Likewise, effective classroom attention requires the integration of arousal regulation, motor inhibition, reward processing, executive control, and future-oriented planning into a stable system capable of sustaining participation in structured learning environments [45,46,47,48].

A developmental timing perspective shifts attention from isolated deficits to patterns of developmental coordination. Children may possess many of the component abilities necessary for successful academic performance, yet those abilities may mature at different rates or become integrated only after formal instruction requires their coordinated use. One system may develop relatively early, another according to an expected trajectory, while a third matures more slowly or is influenced by environmental experience. Academic difficulty may arise not because individual capacities are absent, but because the functional organization of those capacities has not yet achieved the level of coordination required for efficient performance.

Developmental timing may provide a mechanistic link between biological maturation and educational readiness, emphasizing that successful learning depends upon the temporal alignment of interacting neurocognitive systems with the instructional demands encountered during development [49,50,51].

### 2.2. Maturational Asynchrony

Maturational asynchrony refers to differences in the developmental tempo of neurocognitive systems that must ultimately function together to support adaptive behavior. These differences may occur within a single developmental domain, such as language, or across multiple domains, including language, attention, executive function, motor control, emotional regulation, and symbolic learning. Maturational asynchrony is not synonymous with global developmental delay. Developmental variability concerns differences in the maturation of individual neurocognitive systems considered in isolation, whereas maturational asynchrony concerns differences in the relative timing among interacting systems whose coordinated operation is required for successful learning and adaptive behavior. The construct is therefore inherently relational rather than simply dimensional, reflecting the developmental alignment among functionally interdependent systems rather than the level of development attained by any single system. Whereas developmental systems theory emphasizes that cognition emerges from interactions among multiple developing systems, maturational asynchrony refers specifically to differences in the timing and coordination of those interacting systems and proposes that these temporal relationships themselves contribute to learning outcomes. It describes uneven developmental trajectories in which some systems mature at an expected or even accelerated rate, whereas others remain comparatively delayed, unstable, or incompletely integrated. Such uneven developmental profiles are common across learning disabilities and other neurodevelopmental conditions, where strengths and weaknesses frequently coexist rather than reflecting a uniform impairment [28,52].

This distinction is important because academic abilities are integrative achievements rather than isolated cognitive functions. Reading, mathematical reasoning, and self-regulated classroom behavior all depend upon the coordinated operation of multiple component systems. A child may possess an age-appropriate vocabulary yet demonstrate weak phonological segmentation, exhibit intact visual recognition while struggling with efficient grapheme–phoneme integration, display strong curiosity alongside immature inhibitory control, or possess an intuitive sense of quantity without successfully linking those representations to symbolic number systems. In each instance, the educational difficulty is not best understood as the absence of an essential capacity but as an imbalance in the developmental organization of abilities that must ultimately operate as an integrated functional network [49,53].

The effects of maturational asynchrony can be illustrated across the major learning disabilities. In dyslexia, formal reading instruction and increasing exposure to print may occur before phonological awareness, speech-sound discrimination, rapid automatized naming, or language–print integration have achieved sufficient stability. The resulting difficulty reflects not simply impaired reading but inefficient integration between spoken language and written symbols. In ADHD, educational expectations for sustained attention, behavioral inhibition, delayed gratification, task completion, and cognitive flexibility may exceed the developmental readiness of executive, motivational, attentional, motor, and state-regulatory systems. Similarly, in dyscalculia, instruction emphasizing symbolic number concepts may precede the successful integration of approximate magnitude representations, exact quantity processing, spatial number mapping, verbal number knowledge, and working memory [54,55]. Although these conditions differ in their behavioral manifestations, each illustrates how differences in developmental tempo among interacting systems can limit the efficient acquisition of complex academic skills.

Maturational asynchrony also provides a developmental explanation for the inconsistent performance frequently observed in children with learning disabilities. Academic performance often varies substantially across contexts, tasks, and levels of environmental support because the underlying component systems may function reliably under some conditions but not others. A child with dyslexia may decode accurately when sufficient time is available yet struggle when fluent, rapid processing is required, reflecting the dependence of reading fluency on the efficient interaction of phonological processing, rapid naming, visual processing, and serial integration rather than decoding accuracy alone [56,57]. Likewise, a child with mathematical learning difficulties may solve problems successfully using concrete manipulatives while experiencing marked difficulty when required to manipulate abstract symbolic representations, consistent with evidence that symbolic and non-symbolic numerical processing can develop somewhat independently and contribute differently to arithmetic competence [58,59,60]. Children with ADHD often demonstrate adequate attention during highly stimulating or immediately rewarding activities but substantially poorer performance during prolonged, repetitive, or delayed tasks, reflecting the influence of motivational processes, reinforcement contingencies, time-on-task, and intraindividual variability in attentional regulation [61,62,63].

Such variability is frequently interpreted as evidence of inconsistent effort, poor motivation, or oppositional behavior. A developmental timing perspective suggests a different interpretation. Performance may fluctuate because the underlying neurocognitive systems have achieved only partial functional integration and cannot yet support stable performance across changing environmental demands. Under favorable conditions, the available systems may be sufficient to support successful performance; under greater cognitive, temporal, or motivational demands, however, the limitations imposed by maturational asynchrony become increasingly apparent.

From this perspective, poor academic performance reflects more than weakness within an individual cognitive domain. It reflects a mismatch between educational demands and the developmental organization of the systems required to meet those demands. Children are expected to recruit language, perception, executive control, attention, motor regulation, memory, and symbolic processing as coordinated functional networks, yet these systems may not have reached comparable levels of maturity. Dynamic developmental models similarly emphasize that cognition emerges from continuously changing interactions among component systems rather than from isolated abilities operating independently [64,65].

Recognizing maturational asynchrony has important implications for neuroeducation. The educational challenge is not simply to remediate an apparent academic weakness but to facilitate the progressive integration of the underlying systems upon which successful learning depends. This perspective encourages interventions that strengthen the coordination of executive, attentional, motor, language, and symbolic processes while recognizing that these systems continue to influence one another throughout development. Such an approach is consistent with evidence that executive, motor, attentional, and academic functions interact across neurodevelopmental profiles and collectively influence educational achievement over time [66,67].

### 2.3. Developmental Cascades

Developmental cascades describe the process through which relatively small early differences in developmental timing become progressively amplified as children interact with increasingly complex cognitive, social, and educational environments. Rather than remaining confined to the original area of difficulty, early developmental variation alters subsequent learning opportunities, influencing the acquisition of additional skills that depend upon earlier competencies. Over time, these reciprocal influences can produce increasingly divergent developmental trajectories despite relatively modest initial differences [68,69,70].

The cascade concept emphasizes that development is cumulative and self-organizing. Early competencies provide the foundation upon which later abilities are constructed, while early inefficiencies can restrict opportunities for practice, successful performance, positive feedback, and further developmental refinement. Consequently, developmental outcomes reflect not only the initial level of functioning within a particular domain but also the accumulating consequences of interactions between the developing child and the educational environment. Small differences in developmental timing may therefore produce disproportionately large long-term academic consequences because each developmental stage influences the conditions under which subsequent learning occurs [69,71].

Reading acquisition provides one of the clearest examples of developmental cascades. Children who establish efficient phonological representations and accurate grapheme–phoneme correspondences early in development typically experience increasing reading fluency, expanded print exposure, vocabulary growth, improved comprehension, and broader academic knowledge. Conversely, children whose early phonological development remains unstable often read less efficiently, encounter greater difficulty accessing printed information, experience reduced reading practice, and acquire vocabulary and background knowledge more slowly. These cumulative differences extend beyond decoding itself, influencing language development, academic confidence, classroom participation, and educational achievement across multiple subject areas [72,73,74,75].

Comparable cascade processes characterize mathematical development. Early quantity representations provide the basis for symbolic number learning, arithmetic procedures, numerical fluency, and increasingly abstract mathematical reasoning. When foundational numerical concepts develop inefficiently, later mathematical learning becomes progressively more demanding because higher-order skills continue to depend upon incomplete earlier representations. Children may therefore experience growing difficulties not only with arithmetic accuracy but also with mathematical reasoning, problem solving, and confidence in mathematics as curricular demands become increasingly abstract [76,77,78,79].

Developmental cascades are equally evident in attention and executive functioning. Early differences in attentional regulation, inhibitory control, behavioral organization, or working memory influence children’s capacity to engage successfully in classroom instruction, complete assignments, regulate behavior, and benefit from educational feedback. Reduced academic engagement limits opportunities to practice emerging skills, receive corrective instruction, and develop effective learning strategies. These experiences may subsequently influence motivation, self-efficacy, emotional regulation, peer relationships, and long-term educational participation, extending the developmental consequences well beyond the original executive difficulties [80,81,82,83].

Importantly, developmental cascades operate through reciprocal rather than unidirectional influences. Biological maturation shapes children’s responses to educational experiences, while educational experiences modify neural organization through experience-dependent plasticity. Successful learning promotes additional opportunities for practice and increasingly complex cognitive engagement, whereas repeated failure may reduce motivation, increase anxiety, alter teacher expectations, and restrict participation in educational activities that would otherwise support continued development. Thus, developmental trajectories emerge through continuous interactions between intrinsic developmental processes and environmental experiences rather than through maturation alone [84,85,86,87].

The cascade perspective also helps explain why learning disabilities frequently become more apparent over time. During early childhood, relatively modest differences in developmental timing may have limited behavioral consequences because educational demands remain comparatively simple. As formal schooling progresses, however, academic tasks increasingly require the simultaneous coordination of language, executive control, working memory, symbolic processing, attention, motor organization, and self-regulation. Difficulties that were initially subtle may therefore become increasingly visible as the complexity of educational demands exceeds the child’s level of developmental integration [88,89,90].

This developmental interpretation differs fundamentally from models that conceptualize learning disabilities as static impairments. Developmental cascades suggest that educational outcomes remain dynamic because developmental trajectories continue to evolve throughout childhood and adolescence. Early intervention may therefore alter subsequent developmental pathways not merely by improving isolated skills but by modifying the sequence of developmental interactions through which later competencies emerge. Likewise, delayed intervention may allow relatively small developmental differences to accumulate into increasingly complex academic, behavioral, and psychosocial challenges. The developmental timing framework therefore emphasizes both the risks associated with early maturational asynchrony and the opportunities created by interventions that restore or strengthen developmental coordination before cascading effects become firmly established [91,92,93,94].

### 2.4. School-Readiness Mismatch

School-readiness mismatch refers to the discrepancy that arises when educational expectations are determined primarily by chronological age while neurocognitive readiness varies substantially among children because interacting developmental systems mature at different rates. Formal education typically assumes that children entering the same grade possess sufficiently similar cognitive, linguistic, attentional, motor, and self-regulatory capacities to benefit from common instructional experiences. Although this assumption provides a practical basis for organizing educational systems, it overlooks the considerable variability that characterizes normal neurodevelopment. As a result, children may encounter academic expectations before the underlying neurocognitive systems required to meet those expectations have achieved sufficient functional integration [95,96,97].

Developmental variability is particularly evident during the transition into formal schooling. Reading instruction assumes the coordinated availability of phonological awareness, oral language, visual-symbol recognition, attention, working memory, and executive control. Mathematics instruction similarly presupposes stable quantity representations, symbolic number knowledge, language comprehension, spatial processing, and procedural learning. Classroom participation further requires behavioral regulation, sustained attention, cognitive flexibility, emotional regulation, and the capacity to tolerate delayed reinforcement. Because these component systems mature along partially independent developmental trajectories, children of identical chronological age may differ substantially in their readiness to engage successfully with the same educational curriculum [98,99,100,101].

Importantly, school-readiness mismatch does not imply that children are incapable of learning. Rather, it suggests that educational demands may exceed the current level of developmental organization among the interacting systems required for successful learning. Under these circumstances, academic difficulties may emerge not because children lack intellectual capacity, motivation, or educational opportunity, but because instruction requires patterns of neurocognitive coordination that have not yet fully developed. As maturation continues, many children subsequently acquire the capacities that were initially unavailable, whereas others may require targeted educational support to facilitate the integration of those developing systems [102,103,104].

The consequences of school-readiness mismatch extend beyond immediate academic performance. Repeated experiences of difficulty may influence children’s motivation, self-efficacy, classroom engagement, emotional well-being, and relationships with teachers and peers. Early academic struggles can alter instructional experiences by increasing remediation, reducing opportunities for successful participation, or lowering expectations regarding future achievement. These educational responses may inadvertently contribute to the developmental cascades described previously, amplifying relatively modest differences in developmental timing into increasingly persistent academic difficulties [105,106,107].

Recognition of developmental variability has important implications for educational practice. Educational systems have traditionally emphasized age-based progression because chronological age provides a readily observable and administratively practical measure for organizing instruction. However, developmental research consistently demonstrates that neurocognitive maturation shows substantial individual variation even among typically developing children. Consequently, educational readiness may be more accurately conceptualized as a multidimensional developmental profile rather than a fixed age-dependent milestone [108,109,110].

A developmental timing perspective therefore encourages greater flexibility in matching instruction to children’s evolving developmental organization. This does not imply delaying formal education until every developmental system reaches complete maturity, nor does it advocate abandoning age-based educational structures. Rather, it suggests that instruction should recognize developmental heterogeneity by providing multiple pathways through which children can acquire emerging skills while progressively strengthening the underlying neurocognitive systems that support later academic competence. Educational approaches that incorporate graduated instruction, adaptive scaffolding, individualized pacing, and repeated practice may accommodate developmental variability [111,112,113,114,115]. The timing framework makes the more specific prediction that such approaches should be most effective when instructional progression is calibrated to measurable changes in the coordination of the component systems required for the target skill, rather than to chronological age or performance level alone. This prediction is empirically testable by comparing timing-informed instructional decisions with otherwise equivalent adaptive approaches based solely on achievement or age.

**Figure 1:**
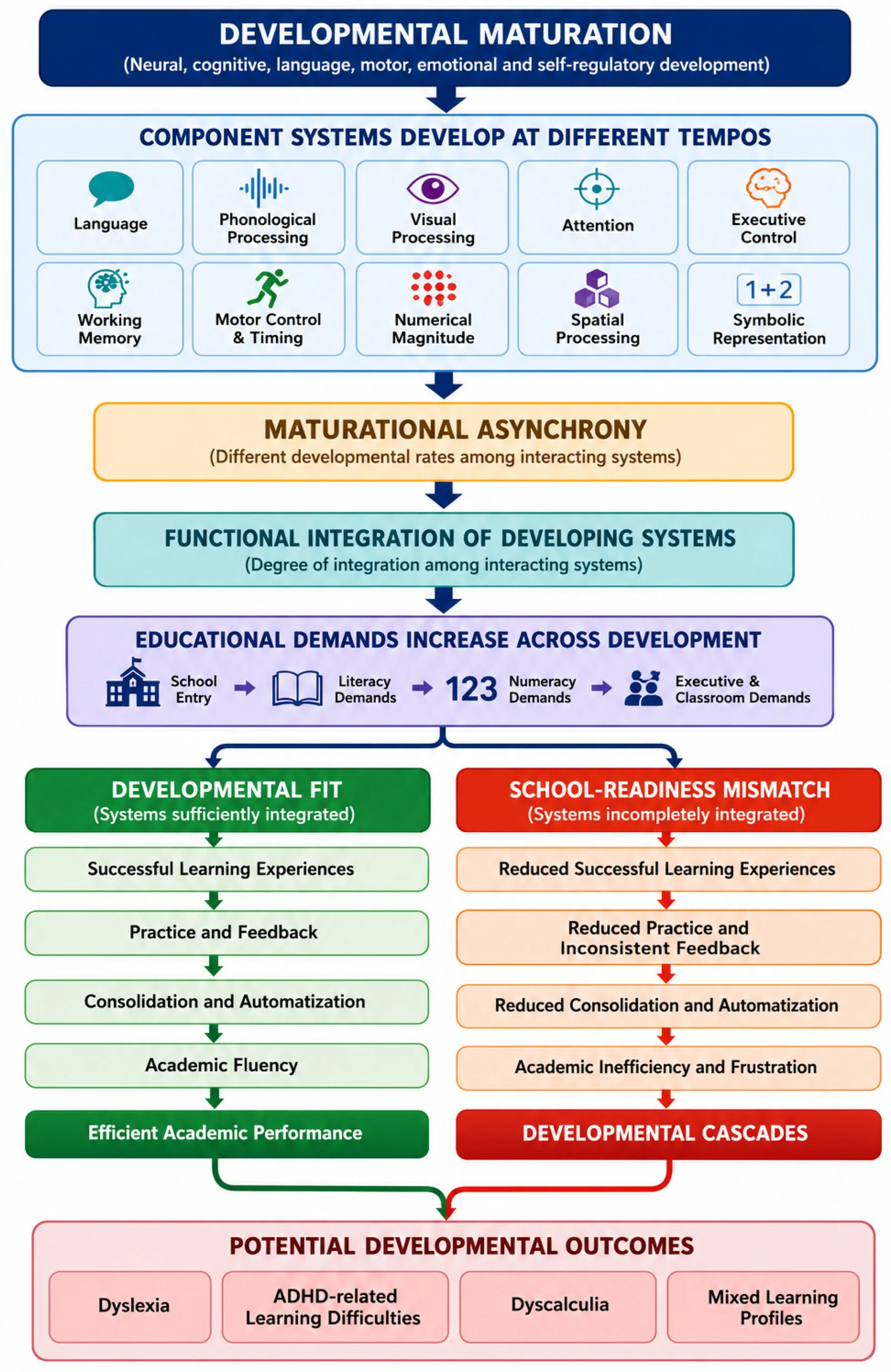
Developmental timing model of learning disabilities. Component neural, cognitive, language, perceptual, motor, numerical, and self-regulatory systems develop along partially independent trajectories and may therefore reach functionally relevant levels of maturity at different times. Maturational asynchrony refers to differences in the developmental rates of interacting component systems, whereas functional integration reflects the degree to which these systems have become sufficiently coordinated to meet emerging task demands. As educational demands increase from school entry through literacy, numeracy, and increasingly complex executive and classroom requirements, the relation between developmental organization and instructional demand may result in either developmental fit or school-readiness mismatch. Developmental fit supports successful learning experiences, practice and feedback, consolidation and automatization, and increasingly efficient academic performance. In contrast, school-readiness mismatch may reduce successful learning opportunities and effective practice, constrain consolidation and automatization, and contribute to academic inefficiency and frustration. These processes may initiate developmental cascades through which initially circumscribed differences become amplified over time. Different configurations and degrees of maturational asynchrony and cross-system integration are proposed to contribute to heterogeneous developmental outcomes, including dyslexia, ADHD-related learning difficulties, dyscalculia, and mixed learning profiles. The pathways depicted are theoretical and probabilistic rather than deterministic or established causal sequences.

Understanding school-readiness mismatch within a developmental timing framework also reframes the interpretation of learning disabilities. Instead of viewing poor academic performance solely as evidence of intrinsic cognitive deficits, developmental timing emphasizes the interaction between children’s evolving neurodevelopmental capacities and the timing of educational expectations. Learning difficulties may therefore arise from the dynamic relationship between biological maturation and instructional demands rather than from fixed impairments within isolated cognitive domains. This perspective provides the conceptual bridge between developmental neuroscience and educational practice that underlies the present theoretical model and establishes the foundation for examining specific learning disabilities through the lens of developmental timing. Figure 1 summarizes the proposed developmental timing framework. It illustrates how variation in developmental timing and cross-system coordination may give rise to maturational asynchrony, developmental cascades, school-readiness mismatch, and ultimately heterogeneous learning profiles.

## 3. Dyslexia as Developmental Asynchrony in Language–Print Integration

The acquisition of culturally defined symbolic systems requires developing cognitive representations to become systematically associated with external symbols, a problem that has been examined explicitly in accounts of symbol grounding and symbol–symbol learning [116]. Dyslexia is typically defined as a neurodevelopmental disorder characterized by persistent difficulties in accurate and/or fluent word recognition, decoding, and spelling despite adequate educational opportunity and intelligence [117]. These reading difficulties have consistently been associated with impairments in phonological processing, although converging evidence indicates that visual, attentional, auditory, linguistic, rapid-naming, working-memory, and executive processes also contribute to individual differences in reading development [56,118,119,120,121]. A developmental timing perspective does not replace these well-established accounts. Rather, it situates them within a broader developmental framework in which successful reading emerges through the progressive functional integration of multiple neurocognitive systems. From this perspective, dyslexia may be understood, in part, as a consequence of developmental asynchrony among the systems responsible for linking spoken language with written symbols [13,14,15]. Specifically, the present framework proposes that variability in the developmental coordination of these interacting systems constitutes an additional, potentially measurable source of individual differences in reading acquisition beyond variation in any single component process.

Reading differs fundamentally from spoken language because it is not a biologically primary capacity. Whereas oral language develops through species-typical interactions among neural maturation, social experience, and communication, reading requires children to establish novel associations between culturally invented visual symbols and pre-existing language systems. Learning to read depends upon the recruitment and reorganization of neural circuits originally supporting visual perception, spoken language, attention, memory, and action rather than upon an evolutionarily specialized reading module [122,123,124,125]. Successful reading emerges as these distributed systems become progressively integrated through repeated interactions with print and language.

The establishment of language–print integration is a developmental process rather than a single instructional event. Children must recognize visual letter forms, discriminate and manipulate phonological units, establish stable grapheme–phoneme correspondences, maintain sequential information in working memory, coordinate speech production, regulate visual attention and eye movements, and progressively automatize decoding through repeated reading experience [56,121,122,126]. Reading proficiency reflects the successful integration of multiple component systems that mature along partially independent developmental trajectories. The efficiency with which these systems become organized during early literacy acquisition determines not only the accuracy of decoding but also the later emergence of fluent and automatic reading [127].

Within this framework, dyslexia is viewed not as the consequence of a single impaired cognitive mechanism but as a developmental disturbance in the integration of language and print. Children may possess many of the individual abilities necessary for reading while nevertheless experiencing difficulty because the systems supporting phonological processing, visual recognition, attention, working memory, and language have not yet achieved the degree of functional organization required for efficient decoding. The developmental timing framework proposes a mechanistic explanation for why reading difficulties often emerge during the transition to formal literacy instruction, why considerable heterogeneity exists among children with dyslexia, and why intervention is most effective when it targets the coordinated development of the underlying neurocognitive systems rather than isolated academic skills alone.

### 3.1. Phonological Development and Language–Print Mapping

The developmental relationship between phonological organization and literacy acquisition provides one of the clearest examples of how maturational timing influences academic learning. Alphabetic writing systems require children to recognize that spoken words can be segmented into discrete phonological units and that visual symbols systematically represent those units [15,117]. The successful acquisition of reading depends not only on learning letter-sound correspondences but also on the developmental availability of phonological representations that are sufficiently stable, accessible, and precisely organized to support rapid mapping between speech and print [15,119].

From a developmental timing perspective, phonological awareness is not simply a prerequisite that is either present or absent. Rather, it reflects the gradual refinement of speech representations through ongoing interactions among auditory perception, language development, speech production, memory, and experience. As these systems mature, children become increasingly able to recognize, manipulate, and retrieve phonological units with the speed and precision required for fluent decoding. Consequently, successful literacy acquisition depends not only on whether phonological awareness develops but also on whether it reaches sufficient developmental maturity when formal reading instruction begins [31,128,129]. This coordination can be examined longitudinally by measuring the relative developmental trajectories of phonological awareness, speech-sound discrimination, grapheme–phoneme learning, decoding accuracy, and reading fluency. The theory predicts that divergence among these trajectories will explain later reading outcomes more accurately than any single component measure alone.

This distinction has important implications for understanding dyslexia. Formal literacy instruction typically assumes that children can rapidly coordinate speech sounds, visual symbols, serial order, articulation, attention, and working memory during the decoding process. These assumptions are rarely explicit within educational practice, yet they place considerable demands on multiple neurocognitive systems that may still be developing. If phonological awareness, speech-sound discrimination, verbal short-term memory, or rapid access to phonological representations remains immature or unstable, instructional demands may exceed the child’s current developmental readiness for efficient language–print integration. The child may correctly identify individual letters, understand that print represents spoken language, and even acquire isolated grapheme–phoneme associations, while continuing to experience slow, effortful, and inconsistent decoding because the underlying systems have not yet achieved sufficient functional integration.

This interpretation remains fully consistent with the central role of phonological processing in contemporary theories of dyslexia while extending those theories developmentally [117,121,128]. Rather than viewing phonological deficits as static characteristics, a developmental timing framework emphasizes that phonological representations must become sufficiently differentiated, rapidly accessible, and efficiently coordinated with visual-symbol processing to support increasingly fluent reading. Reading instruction represents an interaction between developmental readiness and educational demand rather than the simple acquisition of a new academic skill. The same instructional experience may facilitate rapid progress in one child while proving considerably more challenging for another whose underlying language systems have not yet reached comparable levels of developmental organization.

An important consequence of this perspective is that early reading difficulties need not indicate an immutable phonological impairment. Instead, they may reflect developmental variation in the rate at which language and print become functionally integrated. Some children achieve efficient grapheme–phoneme mapping relatively quickly, whereas others require additional developmental experience before phonological, visual, attentional, and mnemonic systems operate together with sufficient precision to support fluent decoding. This interpretation is consistent with evidence that reading acquisition depends on repeated cycles of phonological recoding and self-teaching, through which successful decoding progressively strengthens orthographic representations and promotes increasing automaticity [15,129,130]. When early language–print integration is inefficient, these self-reinforcing learning opportunities become less frequent, increasing the likelihood that relatively modest developmental differences will broaden into more persistent reading difficulties over time.

From this developmental perspective, phonological awareness is best understood as one component of a broader language–print integration system rather than as an isolated cognitive mechanism. Its importance derives not only from its direct contribution to decoding but also from its central role in coordinating auditory, linguistic, visual, articulatory, attentional, and memory processes during the acquisition of literacy. Dyslexia emerges not because phonology alone is impaired, but because the developmental organization of the language–print network has not yet achieved the level of functional integration required by formal literacy instruction.

### 3.2. Rapid Automatized Naming, Auditory Timing, and Orthographic Depth

Although phonological awareness plays a central role in reading acquisition, successful literacy depends upon far more than the representation of speech sounds. Reading requires the rapid coordination of multiple neurocognitive processes under substantial temporal constraints. Children must recognize visual symbols, retrieve phonological representations, maintain serial order, direct visual attention, coordinate articulation, and integrate these operations within fractions of a second during continuous reading. Consequently, developmental differences in the efficiency with which these systems interact may influence reading fluency even when individual component processes appear relatively intact.

Rapid automatized naming (RAN) illustrates this principle particularly well. RAN tasks require children to identify familiar visual stimuli and retrieve their verbal labels quickly and sequentially, engaging visual recognition, lexical access, phonological retrieval, articulation, attentional control, serial processing, and processing speed simultaneously [56,121,128]. Rather than measuring a single cognitive function, RAN provides an index of the efficiency with which multiple neurocognitive systems operate together under temporal constraints [56,120]. Children with dyslexia frequently demonstrate reduced rapid-naming performance, particularly in relation to reading fluency [56,119,121]. From a developmental timing perspective, these findings suggest that the relevant representations may be available but cannot yet be accessed and coordinated with sufficient speed to support automatic reading. This interpretation helps explain why some children eventually achieve accurate decoding while continuing to experience slow, laborious, and effortful reading.

Auditory temporal processing represents another developmental pathway through which language–print integration may be influenced. Spoken language is characterized by rapidly changing acoustic information that requires children to detect subtle temporal differences distinguishing phonemes, syllables, stress patterns, and speech rhythms [128,131]. Efficient auditory processing contributes to the development of stable phonological representations by supporting the accurate encoding of these temporal speech characteristics. When the perception of amplitude rise time, rhythmic structure, or rapidly changing acoustic information is less precise, phonological representations may develop with reduced specificity, making subsequent grapheme–phoneme mapping more demanding [129,130]. Such developmental differences may contribute indirectly to later reading difficulties by influencing the quality of the language representations upon which literacy acquisition depends [128,131].

Importantly, auditory temporal-processing differences are neither universal among children with dyslexia nor specific to the disorder. Contemporary evidence suggests that they represent one of several developmental risk pathways rather than a necessary or sufficient cause of dyslexia [132,133]. This observation is fully consistent with a developmental timing framework, which does not assume that all children follow the same developmental trajectory. Instead, different combinations of auditory, phonological, attentional, visual, and language-related vulnerabilities may produce similar behavioral outcomes because each ultimately influences the efficiency with which spoken language becomes integrated with print.

The influence of orthographic depth further illustrates that developmental timing reflects an interaction between child characteristics and educational context. Writing systems differ substantially in the consistency with which visual symbols correspond to spoken language. In relatively transparent orthographies, grapheme–phoneme relationships are highly regular and can be acquired with comparatively fewer learning trials. In deeper orthographies, such as English, successful reading requires the progressive integration of phonological, orthographic, morphological, and lexical knowledge across a more extended developmental period. Cross-linguistic and meta-analytic evidence demonstrates that orthographic depth modifies the behavioral expression of developmental dyslexia, suggesting that comparable neurodevelopmental vulnerabilities may become more or less apparent depending upon the temporal and cognitive demands imposed by the writing system itself [13,14].

Rapid automatized naming is therefore interpreted less as an isolated deficit than as an indicator of the efficiency with which distributed language–print systems have become developmentally coordinated under temporal constraints.

Viewed collectively, rapid automatized naming, auditory temporal processing, and orthographic depth illustrate a common developmental principle. Reading proficiency depends not only on the integrity of individual cognitive processes but also on the efficiency with which multiple neurocognitive systems become integrated under the temporal demands imposed by literacy acquisition. Different developmental pathways may converge on similar reading difficulties because they influence different components of the same evolving language–print network. Dyslexia is consequently better understood as a heterogeneous developmental disorder arising from multiple interacting trajectories rather than as the expression of a single impaired mechanism. Accordingly, the theory predicts that identical developmental profiles may produce different behavioral manifestations across orthographies because educational demands differ in the timing and complexity of language–print integration.

### 3.3. Orthographic Learning, Visual Attention, and Motor Contributions

The transition from accurate decoding to fluent reading represents a major developmental milestone in literacy acquisition. Early reading is typically characterized by effortful grapheme–phoneme conversion, whereas skilled reading depends increasingly on the rapid recognition of familiar orthographic patterns and whole-word representations. This transition is not simply a consequence of repeated exposure to print but reflects the progressive consolidation of language–print associations through successful reading experiences. Each accurate decoding event provides an opportunity to strengthen connections between orthographic forms, phonological representations, and lexical knowledge, gradually reducing the cognitive effort required for word recognition [121,122].

This developmental progression is well described by the self-teaching hypothesis, in which repeated phonological recoding enables the establishment of increasingly stable word-specific orthographic representations [129,130]. Orthographic learning depends upon successful interactions among phonological, visual, linguistic, attentional, and memory systems rather than on visual experience alone. This relationship is also testable. Children showing earlier coordination among decoding accuracy, orthographic learning, and reading fluency should demonstrate steeper subsequent growth in reading than children whose component systems remain developmentally asynchronous despite similar initial decoding ability.

When early decoding is slow, inaccurate, or inconsistent, opportunities for efficient self-teaching are reduced, limiting the consolidation of orthographic knowledge and delaying the emergence of automatic word recognition [122]. Children may consequently remain dependent on laborious phonological decoding long after their peers have transitioned toward increasingly automatic reading, with subsequent reductions in reading fluency, comprehension, and print exposure [134].

Visual attention contributes to this developmental process by regulating how written information is sampled and integrated during reading. Efficient reading requires children to allocate attention across letters and letter clusters, preserve serial order, coordinate saccadic eye movements, and integrate visual information with simultaneously activated phonological and lexical representations [135,136]. These operations occur continuously during reading and depend upon the rapid interaction of visual, attentional, and linguistic systems. Difficulties in visual attention or serial visual processing do not necessarily constitute alternative explanations for dyslexia; rather, they may increase the demands placed on an already developing language–print network by reducing the efficiency with which orthographic information is extracted and integrated [137,138].

Motor and articulatory processes provide an additional, often underappreciated, contribution to literacy development. Phonological awareness is closely linked to speech production, subvocal rehearsal, articulatory planning, and the reciprocal interactions between auditory and motor representations of speech [56,128]. Reading aloud further requires the rapid integration of visual input, lexical access, phonological retrieval, motor planning, and articulation into a coordinated behavioral sequence [56,120]. Even when overt motor impairments are absent, developmental differences in the efficiency with which perceptual and articulatory systems interact may influence the speed with which decoding becomes automatic. Fluency reflects not only accurate language–print mapping but also the progressive refinement of perception–action coupling throughout literacy development [56,119,121].

Working memory similarly contributes to the transition from effortful decoding to fluent reading. During early literacy acquisition, children must temporarily maintain phonological information while integrating sequential visual input, retrieving lexical representations, and monitoring comprehension. As orthographic representations become increasingly stable, the burden placed on working memory gradually decreases because familiar words can be recognized with minimal conscious analysis. Conversely, inefficient orthographic learning prolongs reliance on controlled decoding, increasing working-memory demands and limiting the cognitive resources available for higher-order language comprehension. Difficulties in comprehension may emerge not because semantic or linguistic abilities are fundamentally impaired but because cognitive resources remain occupied by lower-level decoding processes [139].

Viewed from a developmental timing perspective, these interacting processes illustrate that reading fluency is an emergent property of progressively integrated neurocognitive systems rather than the product of any single cognitive ability. Orthographic learning, visual attention, motor coordination, articulation, and working memory each contribute to literacy because they participate in the repeated cycles of successful language–print integration through which reading becomes increasingly efficient. Variability in any one of these systems may influence the pace of literacy acquisition, while differences across several systems may combine to produce the heterogeneous developmental profiles observed in dyslexia. Reading difficulties arise not from isolated weaknesses in individual component processes but from variation in the developmental organization of the broader neurocognitive network supporting fluent literacy.

### 3.4. Developmental Emergence, Heterogeneity, and Educational Implications

A developmental timing framework helps explain why dyslexia typically becomes apparent during the first years of formal schooling, when children are expected to transform developing oral-language abilities into efficient and increasingly automatic print–speech integration [121,128,140]. Before school entry, communication is supported by multiple contextual cues, including gesture, facial expression, prosody, shared attention, and situational context, allowing children to comprehend and produce spoken language despite considerable variation in underlying neurocognitive development. Formal literacy instruction substantially changes these demands by requiring rapid, explicit, and repeated coordination between written symbols and spoken language with progressively decreasing reliance on contextual support [15,31,129].

From this perspective, reading difficulties often become educationally apparent when formal instruction begins rather than emerging suddenly at that point. Schooling reveals developmental differences that were previously accommodated within natural language environments. As instructional demands increase, children whose phonological, visual, attentional, auditory, articulatory, and working-memory systems have become sufficiently integrated generally acquire decoding with increasing efficiency. In contrast, children whose underlying developmental trajectories remain asynchronous may experience reading as disproportionately slow, effortful, and cognitively demanding because the functional organization required for fluent language–print integration has not yet been established [56,119,120]. The onset of formal reading instruction represents a developmental transition at which maturational differences become educationally consequential rather than a point at which dyslexia first develops.

This developmental perspective also provides a framework for understanding the marked heterogeneity observed among children with dyslexia. Some children present with prominent phonological weaknesses, others exhibit reduced rapid naming or reading fluency, whereas others demonstrate broader language, attentional, executive, or working-memory difficulties. Longitudinal studies continue to indicate that no single cognitive profile adequately characterizes all individuals with dyslexia and that developmental trajectories differ substantially across children [119]. Such variability is expected within a developmental timing framework because different neurocognitive systems mature along partially independent trajectories. Reading outcomes depend not only on which systems show developmental variation but also on the magnitude of those differences, the interactions among them, and the educational demands encountered during literacy acquisition.

Importantly, this perspective shifts the emphasis from identifying a single causal deficit toward understanding developmental profiles. Dyslexia is better conceptualized as an emergent consequence of variation in the developmental organization of the language–print network than as a fixed impairment in one cognitive process. This interpretation accommodates the well-established importance of phonological processing while simultaneously incorporating evidence implicating rapid naming, auditory processing, orthographic learning, visual attention, working memory, executive functions, and language development as interacting contributors to reading acquisition. Rather than competing explanations, these mechanisms represent complementary pathways through which developmental variation may influence literacy outcomes.

The developmental timing framework also has important implications for educational practice. Intervention should not be viewed simply as increasing exposure to print or providing additional opportunities for decoding practice. Effective intervention aims to strengthen the component processes that support efficient language–print integration, including phonological awareness, auditory discrimination, rapid lexical retrieval, orthographic learning, visual attention, verbal working memory, reading fluency, and the coordinated interaction of these processes during literacy acquisition [15,120,121,140]. The framework further predicts that interventions may be most effective when they are introduced during periods in which the relevant component systems remain sufficiently plastic to strengthen their functional coordination before secondary developmental cascades become established. Equally important is the timing of intervention. Providing appropriate support while these systems remain highly plastic may reduce developmental divergence, strengthen reciprocal interactions among emerging reading processes, and improve subsequent academic outcomes.

Viewed as a whole, dyslexia illustrates a broader principle of neurodevelopment. Academic learning depends upon the progressive functional integration of multiple neurocognitive systems whose developmental trajectories are neither identical nor perfectly synchronized. Reading difficulties arise when increasing educational demands exceed the current level of developmental organization within the language–print network. Dyslexia represents not merely a disorder of reading but an example of how variation in developmental timing can shape learning trajectories more generally. This perspective provides a conceptual bridge to other learning disabilities, in which different neurocognitive systems and different patterns of maturational asynchrony underlie distinct academic profiles.

### 3.5. Synthesis: Dyslexia as a Developmental Timing Disorder

The developmental timing framework presented here does not reject established theories of dyslexia. Rather, it provides a broader developmental context within which these theories can be understood as describing different components of an evolving literacy system. Phonological processing, rapid automatized naming, auditory temporal processing, orthographic learning, visual attention, working memory, language development, and motor speech each contribute to reading because they participate in the progressive organization of the language–print network. Their relative importance may differ across individuals, but successful reading ultimately depends on the emergence of efficient interactions among these component systems rather than on the integrity of any single process in isolation [117,119,120,121,128,129,131].

From this perspective, dyslexia is best understood as an expression of developmental asynchrony within a distributed neurocognitive system. Individual components of the reading network may be largely intact, yet literacy acquisition may remain inefficient because their developmental trajectories have not converged sufficiently to support fluent language–print integration. The behavioral manifestations of dyslexia reflect the cumulative consequences of developmental divergence rather than the failure of one isolated cognitive mechanism. This interpretation accommodates both the considerable heterogeneity observed across individuals and the multiple developmental pathways through which reading difficulties may arise [119,120,121].

Importantly, developmental timing is not synonymous with developmental delay. Children with dyslexia do not simply progress more slowly along a uniform developmental pathway. Rather, different neurocognitive systems may mature at different rates, resulting in changing patterns of strengths and weaknesses across development. Consequently, developmental profiles may evolve as literacy experience, neuroplasticity, educational intervention, and continued brain maturation progressively reshape interactions among the component systems supporting reading. This dynamic perspective is consistent with longitudinal evidence demonstrating substantial variability in developmental trajectories and response to intervention [119,140].

Viewing dyslexia through a developmental timing lens also emphasizes that literacy acquisition represents a reciprocal process. Reading instruction modifies the neural systems that support reading, while the child’s current developmental organization simultaneously influences the effectiveness of instruction. Development emerges through continuous interactions between biological maturation and educational experience rather than through either process alone [10,11,84,85,86].

The framework generates several empirical predictions. Longitudinal studies should demonstrate that measures of developmental coordination among phonological, orthographic, attentional, and language systems predict later reading achievement beyond isolated component measures. More specifically, dyslexia is expected to be associated not simply with weak phonological processing but with measurable divergence in the developmental trajectories linking phonological awareness, rapid naming, orthographic learning, and reading fluency. Similarly, interventions timed to periods of emerging cross-system coordination should produce greater and more durable improvements than interventions based solely on chronological age or current reading performance. Confirmation of these predictions would distinguish a developmental timing account from conventional component-deficit models.

Dyslexia illustrates a general principle that extends beyond literacy. Learning difficulties frequently arise when educational demands exceed the level of functional organization achieved by the underlying neurocognitive systems at a particular point in development. Although the specific systems differ across disorders, the developmental principle remains the same: successful learning depends on the progressive integration of interacting neural, cognitive, perceptual, linguistic, and behavioral processes. Dyslexia is interpreted as one manifestation of a broader developmental timing model that may also help explain the emergence of other learning disabilities.

The following section extends this framework to attention-deficit/hyperactivity disorder (ADHD), where developmental asynchrony primarily affects attentional regulation, executive control, behavioral organization, and state regulation rather than language–print integration. Examining ADHD within the same developmental framework allows common developmental principles to be distinguished from disorder-specific mechanisms while further illustrating how variation in maturational timing shapes diverse learning trajectories.

## 4. ADHD as Developmental Asynchrony in Executive-Regulatory Integration

ADHD is among the most common neurodevelopmental conditions affecting school-aged children and is associated with significant difficulties in academic achievement, classroom participation, and self-regulated learning [66,138]. Traditionally, ADHD has been conceptualized as a disorder of inattention, hyperactivity, impulsivity, or executive dysfunction [16,138,139]. These descriptions accurately characterize the clinical manifestations of the disorder but provide only a partial account of the developmental processes that underlie them. A developmental timing perspective instead asks how the neurocognitive systems responsible for regulating behavior become organized across development and how variation in their maturation influences learning.

Unlike dyslexia, in which the principal developmental challenge concerns the integration of spoken language with written symbols, ADHD primarily involves the progressive organization of executive and regulatory systems that enable children to sustain attention, inhibit inappropriate responses, maintain goals in working memory, regulate motivation and arousal, sequence behavior, and adapt performance to changing environmental demands. These capacities emerge gradually through interactions among distributed neural networks rather than through the maturation of a single executive control system. Consequently, developmental variation may be expressed as differences in the timing and efficiency with which these systems become functionally integrated.

From this perspective, ADHD may be conceptualized as involving developmental differences in executive and regulatory organization rather than solely as a deficit of attention or behavioral control. Learning difficulties may emerge, in part, because classroom activities are inherently organized in time, requiring children to coordinate attention, motivation, executive control, motor behavior, and self-regulation across increasingly complex sequences of action. When these systems mature asynchronously or remain insufficiently integrated, the temporal demands of schooling may exceed the child’s current regulatory capacity. The following sections examine how variation in developmental timing may contribute to executive functioning, temporal processing, motivational regulation, classroom performance, and the educational consequences of ADHD.

### 4.1. Executive and Regulatory Development

Attention-deficit/hyperactivity disorder (ADHD) is typically characterized by developmentally inappropriate levels of inattention, impulsivity, hyperactivity, and executive dysfunction that interfere with academic, social, and everyday functioning [138,139]. Predominantly inattentive presentations, historically referred to as attention-deficit disorder (ADD), may be less behaviorally disruptive than combined or hyperactive presentations but are often associated with equally significant educational difficulties [66,138]. Although these clinical descriptions accurately characterize the observable manifestations of ADHD, they provide only a partial explanation of the underlying developmental processes. From a developmental timing perspective, ADHD may be understood not simply as a disorder of attention or behavioral control, but as altered maturation and functional integration of the regulatory systems that organize behavior across time.

Successful self-regulation requires the progressive coordination of multiple neurocognitive processes that develop over childhood and adolescence. These include sustained attention, inhibitory control, working memory, executive planning, motivational regulation, motor control, and arousal regulation, each of which contributes to the ability to delay responses, sustain effort, sequence actions, monitor performance, and organize present behavior toward future goals [16,66,139]. These processes do not mature simultaneously. Rather, they follow partially independent developmental trajectories that become increasingly integrated through ongoing brain maturation and experience. Difficulties may arise when this integration remains incomplete while educational environments increasingly demand coordinated self-regulation.

This developmental interpretation is consistent with the major theoretical accounts of ADHD. Models emphasizing behavioral inhibition, executive dysfunction, delay aversion, motivational dysregulation, state regulation, and temporal processing each identify important components of the disorder [16,18,19,140]. Rather than viewing these models as competing explanations, a developmental timing framework interprets them as describing different aspects of the same evolving regulatory system. Behavioral inhibition reflects the capacity to delay or suppress inappropriate responses; executive functions support the maintenance, updating, sequencing, and monitoring of goal-directed behavior; motivational systems determine the influence of immediate and delayed rewards; and state-regulatory mechanisms maintain levels of arousal appropriate for current task demands. Academic functioning depends upon the coordinated operation of all of these systems rather than upon any one process in isolation.

The importance of this coordination becomes particularly evident in educational settings. Classroom learning is inherently organized in time. Children are expected to wait before responding, initiate tasks when instructed, sustain attention over extended periods, shift flexibly between activities, remember delayed instructions, inhibit competing responses, and complete multistep assignments whose rewards may not be immediately apparent [16,141,142]. These expectations require the functional integration of executive control, attentional regulation, working memory, motivational processes, motor control, and arousal regulation [16,66,140,141]. When these systems develop asynchronously, classroom expectations may exceed the child’s current regulatory capacity. Consequently, educational difficulties may arise not simply from difficulties in attention but also from insufficient coordination among attention, action, motivation, and self-regulation relative to the temporal demands of learning [63,141,143].

Viewed in this way, the framework conceptualizes ADHD as involving developmental differences in executive and regulatory organization rather than a deficit in any single cognitive function. The central issue is not merely whether children can attend, inhibit, or plan, but whether these processes have become sufficiently integrated to support adaptive behavior over time. This developmental perspective provides a conceptual framework for understanding why children with ADHD often display marked variability across contexts, why symptoms change across development, and why educational performance depends as much on the temporal organization of regulatory processes as on the integrity of attention itself.

### 4.2. Temporal Organization of Attention, Executive Control, and Motivation

One of the defining characteristics of ADHD is difficulty organizing behavior in relation to time. Children may struggle to sustain attention across extended intervals, delay responses, estimate task duration, maintain goals over time, or adjust behavior according to future consequences. Meta-analytic evidence indicates that temporal processing difficulties are common in children and adolescents with ADHD and may persist into adulthood [20,142]. These findings suggest that ADHD is not solely a disorder of attention but also involves developmental differences in the mechanisms that coordinate cognition and behavior over time.

Temporal processing encompasses a range of related abilities, including duration discrimination, time estimation, time reproduction, temporal anticipation, and the use of temporal information to guide behavior. These abilities support everyday classroom functioning by allowing children to pace their work, anticipate transitions, regulate effort, and maintain task goals over appropriate intervals. Difficulties in temporal processing may influence learning even when basic intellectual abilities remain intact because successful classroom participation requires continuous alignment between internal regulatory processes and externally imposed schedules, task durations, and instructional sequences. Executive functions provide the cognitive mechanisms through which behavior is organized across time. Within the present framework, developmental coordination among executive control, temporal processing, motivational regulation, and state regulation constitutes the primary construct of interest, rather than the level of impairment observed in any single component process.

Working memory maintains task goals during delays, inhibitory control suppresses competing responses, cognitive flexibility enables adaptive shifts between tasks or strategies, and planning organizes actions toward future outcomes [16,66]. These executive processes mature gradually throughout childhood and adolescence as distributed neural networks supporting frontal, parietal, subcortical, and cerebellar functions become increasingly integrated. Consequently, executive control is best understood as an evolving developmental capacity rather than a fixed cognitive skill.

From a developmental timing perspective, executive difficulties in ADHD are proposed to reflect not simply impairment in individual executive functions but also insufficient coordination among these functions in supporting efficient self-regulation. A child may understand classroom expectations yet struggle to maintain goal representations, inhibit distractions, organize sequential actions, or monitor performance consistently across time. The result is often marked variability in academic performance, with successful task completion depending heavily on external structure, immediate feedback, novelty, or adult support rather than on stable internal regulation [16,63,142,144].

Motivational regulation further illustrates the temporal nature of ADHD. School learning frequently requires sustained effort in the absence of immediate reinforcement, with the benefits of reading, mathematics, writing, or studying emerging only after repeated practice. Numerous studies have demonstrated that individuals with ADHD tend to discount delayed rewards more steeply than typically developing peers, showing a greater preference for smaller immediate rewards over larger delayed outcomes, although the magnitude of this effect varies across developmental stages and experimental paradigms [141,143,145]. Rather than reflecting reduced motivation, per se, these findings suggest developmental differences in the efficiency with which future outcomes regulate present behavior. Executive control and motivational regulation are closely interconnected. Working memory allows future goals to remain active during ongoing activity, inhibitory control suppresses behaviors that compete with those goals, and motivational systems determine the degree to which delayed outcomes influence present effort. Performance-based rewards may also modify feedback-related processing in children with ADHD, further demonstrating the interaction between motivational context and regulatory function [146]. When these processes are insufficiently integrated, immediate environmental events, novel stimuli, or transient impulses may exert greater influence over behavior than long-term academic objectives [16,62,141]. The child often knows what should be done but experiences difficulty sustaining the regulatory processes necessary to translate intention into consistent action.

This interpretation also helps explain why children with ADHD frequently display substantial fluctuations in performance. Tasks that are novel, highly engaging, externally structured, or associated with immediate reinforcement may be completed successfully, whereas repetitive, weakly reinforced, or self-directed activities often prove considerably more difficult [62,147,148]. Such variability does not indicate inconsistent motivation or selective effort. Instead, it reflects the interaction between developmental differences in executive regulation and the temporal characteristics of the learning environment.

Viewed collectively, temporal processing, executive control, and motivational regulation represent complementary components of a broader self-regulatory system. The framework proposes that ADHD-related difficulties may reflect not only impairment in individual mechanisms but also incomplete developmental integration among these systems during periods when educational environments increasingly depend upon their coordinated operation. This developmental interpretation accommodates the diversity of contemporary ADHD theories while emphasizing the common principle that successful learning depends upon the progressive organization of cognition, motivation, and behavior across time.

### 4.3. State Regulation, Motor Timing, and Classroom Performance

The successful regulation of behavior depends not only on executive control and motivation but also on the child’s ability to maintain an appropriate level of physiological and cognitive activation over time. State-regulation theories propose that ADHD involves developmental differences in the mechanisms responsible for maintaining optimal levels of arousal and effort during goal-directed activity [140,148]. These mechanisms enable children to adjust their level of alertness in response to changing environmental demands, sustain engagement during prolonged tasks, and regulate effort as activities become more or less challenging. When state regulation is unstable, attention and behavioral control may fluctuate despite intact knowledge of task requirements.

The educational consequences of altered state regulation become particularly evident in classroom environments, where children are expected to maintain attention and effort over extended periods while adapting to varying instructional demands. Long periods of passive listening, repetitive practice, and delayed feedback and low levels of external stimulation place considerable demands on self-regulatory systems [62,143,149]. Children with ADHD may alternate between periods of appropriate engagement and periods of under-arousal, excessive activity, or mental disengagement as they attempt to regulate their internal state in response to the temporal characteristics of the classroom [140,148]. From a developmental timing perspective, these fluctuations are proposed to reflect difficulties maintaining synchronization between internal regulatory processes and the external pace of learning rather than simple failures of attention or motivation.

This framework also helps explain the predominantly inattentive presentation of ADHD. Unlike children whose difficulties are expressed through overt hyperactivity or impulsive behavior, those with inattentive presentations often exhibit slow task initiation, inconsistent alertness, reduced mental persistence, and difficulties maintaining internally generated sequences of thought and action [63,140,143,148]. These children may appear quiet, compliant, or withdrawn while nevertheless experiencing substantial difficulty following instruction, maintaining the continuity of learning, or independently organizing complex academic activities [62,141]. Their educational challenges may arise in part from instability in the regulatory processes that sustain attention, effort, and goal-directed behavior across time rather than from an absence of intelligence or motivation.

Motor behavior represents another important component of regulatory timing. Hyperactivity and impulsivity are typically described as excessive movement or premature responding, yet they also reflect the developmental organization of action within temporal and environmental constraints [16,150]. Effective classroom participation requires children to wait before responding, inhibit movements that interfere with ongoing activities, coordinate actions with teacher instructions, and participate in socially appropriate turn-taking. Difficulties in these behaviors suggest challenges in aligning motor output with the temporal demands of both the physical and social environment rather than simply an increase in motor activity.

These behavioral characteristics are consistent with the functions of distributed cortico-subcortical networks, including basal ganglia–cortical circuits that contribute to motor control, behavioral sequencing, reward processing, and executive regulation [150]. These neural systems support the coordination of movement, cognition, and motivation during goal-directed behavior and play a central role in the timing demands imposed by classroom learning. Difficulties in coordinating these systems may be expressed as impulsive responding, premature actions, interruptions of ongoing activity, or excessive movement at developmentally inappropriate times. Such behaviors are educationally important because they disrupt not only task completion but also the sequential organization of learning, classroom participation, and social interaction.

State regulation, attentional stability, and motor timing illustrate how ADHD may be conceptualized as involving differences in dynamic behavioral organization rather than solely as a static deficit of attention. Children may demonstrate appropriate performance under conditions that provide immediate feedback, high levels of engagement, or substantial external structure, yet struggle when learning depends on internally maintained regulation across extended periods. Consequently, the framework proposes that the behavioral variability characteristic of ADHD may reflect fluctuations in the efficiency with which executive, motivational, arousal, and motor systems interact during ongoing activity rather than inconsistency in effort or willingness to learn.

From a developmental perspective, these findings reinforce the view that educational performance depends on the continuous coordination of multiple regulatory systems operating over time. Difficulties in attention, state regulation, and motor control may represent interacting manifestations of a broader developmental process involving the progressive functional integration of the neurocognitive systems that organize adaptive behavior. he framework proposes that ADHD may become increasingly educationally significant when this integration remains incomplete while classroom environments increasingly require stable self-regulation, sustained attention, and precisely timed behavioral responses.

### 4.4. Developmental Emergence, Educational Mismatch, and Cascades

A developmental timing framework helps explain why ADHD often becomes increasingly apparent as children enter and progress through formal education. Although differences in attention, activity level, or self-regulation may be evident during the preschool years, many young children function adequately in environments characterized by frequent adult guidance, immediate feedback, flexible routines, and opportunities for movement [16,139,151]. Formal schooling substantially alters these developmental demands. Children are increasingly expected to regulate their own behavior, sustain attention over longer periods, delay gratification, organize multistep activities, and maintain consistent performance under externally imposed schedules. Consequently, educational settings place growing demands on executive and regulatory systems that are themselves still undergoing prolonged development.

From this perspective, ADHD does not suddenly emerge when children enter school. Rather, the transition to formal education exposes developmental differences that previously had limited functional consequences. As expectations for sustained attention, behavioral inhibition, planning, working memory, and independent task management increase, children whose executive and regulatory systems remain developmentally asynchronous may experience an increasing mismatch between their current neurodevelopmental organization and the temporal structure of classroom learning [62,148,151]. The framework proposes that ADHD may become increasingly educationally significant as developmental variation intersects with progressively more complex environmental demands, rather than because executive dysfunction suddenly appears.

This developmental mismatch frequently initiates cascading effects that extend well beyond the primary symptoms of ADHD. Children who miss instructions, initiate tasks slowly, fail to sustain attention, or abandon activities before completion often receive fewer opportunities for repeated practice and skill consolidation [62,143,152]. Because many academic skills, including reading fluency, written expression, arithmetic fact retrieval, and procedural learning, depend upon repeated, well-organized practice, disruptions in executive regulation may gradually influence academic development across multiple domains [153]. What initially appears as difficulty sustaining attention may broaden into more generalized educational underachievement through cumulative reductions in effective learning opportunities.

Developmental cascades also extend beyond academic performance. Repeated experiences of failure, negative feedback, incomplete work, and classroom correction may influence children’s developing beliefs about their own competence and capacity to succeed. Over time, these experiences can reduce academic motivation, self-efficacy, and school engagement while also affecting relationships with teachers, parents, and peers [62]. These secondary consequences are not inevitable, but they illustrate how relatively modest differences in executive and regulatory development may become amplified through continuing interactions between the child and the educational environment.

Importantly, a developmental timing framework emphasizes that these cascades are dynamic rather than deterministic. Executive and regulatory systems continue to mature throughout childhood and adolescence, and developmental trajectories remain responsive to experience, educational support, and intervention. Improvements in classroom structure, task organization, external scaffolding, behavioral supports, and motivational strategies may strengthen the functional integration of attention, executive control, self-regulation, and goal-directed behavior while reducing the accumulation of secondary academic and psychosocial difficulties [154]. Early identification is valuable not because it prevents an inevitable developmental course but because it provides opportunities to modify developmental trajectories while neurocognitive systems retain substantial plasticity.

Viewed from this perspective, ADHD illustrates how educational outcomes emerge through continuous interactions between neurodevelopment and learning environments. Academic success depends not only on children’s cognitive abilities but also on whether the developmental organization of executive, attentional, motivational, motor, and state-regulatory systems is sufficient to meet the temporal demands imposed by schooling. When these demands exceed the child’s current level of functional integration, educational difficulties become increasingly apparent and may broaden through developmental cascades. Conversely, educational environments that appropriately scaffold self-regulation can reduce developmental mismatch, support adaptive learning trajectories, and promote progressively more effective executive functioning over time.

### 4.5. Synthesis: ADHD as a Developmental Timing Disorder

The developmental timing framework proposed here does not replace established theories of ADHD but provides a broader developmental context within which they can be understood. Models emphasizing behavioral inhibition, executive dysfunction, delay aversion, motivational dysregulation, state regulation, temporal processing, and altered reward sensitivity each identify important features of ADHD. Rather than representing competing explanations, these perspectives describe interacting components of a developing self-regulatory system whose successful integration is essential for adaptive learning and behavior. The diversity of theoretical models reflects the complexity of the regulatory networks involved rather than the existence of mutually exclusive causal mechanisms [16,18,19,140,142,148].

From this perspective, the framework conceptualizes ADHD as involving differences in developmental organization within distributed executive and regulatory systems. Attention, inhibitory control, working memory, motivation, arousal, motor regulation, and behavioral sequencing each contribute to goal-directed behavior because they operate as components of an integrated neurocognitive network. Individual systems may function adequately in isolation, yet academic performance may remain inconsistent when their developmental trajectories have not converged sufficiently to support stable self-regulation across changing environmental demands. The framework proposes that the characteristic variability of ADHD may reflect developmental differences in the organization of executive-regulatory networks rather than isolated deficits in attention or impulse control [16,139,150].

Importantly, developmental asynchrony should not be equated with simple developmental delay. Executive and regulatory systems mature over extended periods and follow partially independent developmental trajectories that continue to evolve throughout childhood and adolescence. Consequently, the behavioral profile of ADHD is dynamic rather than static. As brain maturation, educational experience, environmental structure, and intervention reshape interactions among executive, motivational, attentional, and motor systems, patterns of strengths and weaknesses may also change. This developmental perspective helps explain the marked variability in symptom expression across individuals, developmental stages, and learning environments [62].

The developmental timing framework also emphasizes the reciprocal relationship between neurodevelopment and education. Classroom experiences influence the maturation of executive and self-regulatory processes, while the child’s current level of regulatory organization simultaneously determines the effectiveness of educational instruction. Learning emerges through continuous interactions between biological maturation and environmental experience rather than through either process alone [10,11,84,85,86]. Educational environments that provide appropriate structure, timely feedback, graduated independence, and opportunities for successful self-regulation may strengthen the functional integration of executive systems and reduce the accumulation of secondary academic and psychosocial difficulties [154]. Importantly, the present framework predicts not simply that structured interventions are beneficial, but that their effectiveness depends on their alignment with the child’s evolving developmental organization. Interventions introduced when executive, attentional, motivational, and state-regulatory systems are becoming functionally coordinated should produce greater and more durable improvements than equally intensive interventions delivered without regard to developmental timing. This prediction distinguishes the developmental timing framework from approaches that individualize instruction primarily on the basis of chronological age, diagnostic category, or current academic performance alone.

The developmental timing framework also generates specific empirical predictions. Longitudinal studies should demonstrate that measures of developmental coordination among executive control, sustained attention, motivational regulation, temporal processing, and state regulation predict later academic functioning more accurately than isolated measures of any single regulatory domain. Likewise, interventions designed to strengthen the functional coordination of these interacting systems and introduced during periods of emerging developmental integration should produce broader and more durable educational benefits than interventions guided solely by chronological age, diagnostic category, or current symptom severity. If these predictions are not supported, the developmental timing framework would receive less empirical support than conventional component-based accounts. Conversely, consistent longitudinal evidence would support the hypothesis that maturational coordination contributes independently to educational outcomes.

More broadly, ADHD illustrates the same developmental principle identified in dyslexia, despite involving different neurocognitive systems. Within the present framework, educational difficulties in dyslexia are proposed to relate primarily to developmental variation in language–print integration, whereas those associated with ADHD are proposed to relate principally to variation in executive and regulatory organization. In both conditions, however, learning becomes vulnerable when increasing educational demands exceed the current level of functional integration achieved by the underlying neurodevelopmental systems. The specific mechanisms differ, but the developmental principle remains the same.

The following section extends this framework to developmental dyscalculia, where the principal challenge involves the progressive integration of quantity representation, symbolic number processing, visuospatial cognition, working memory, and executive control. Examining dyscalculia within the same developmental timing framework further illustrates how distinct patterns of maturational asynchrony may produce different learning profiles while reflecting common developmental principles governing the emergence of academic competence.

## 5. Dyscalculia as Developmental Asynchrony in Number-Symbol Integration

Mathematical competence develops through the progressive coordination of multiple neurocognitive systems rather than through the maturation of a single numerical ability. Children must integrate intuitive representations of quantity with language, symbolic notation, visuospatial organization, working memory, executive control, procedural learning, and increasingly abstract mathematical concepts. These systems emerge along partially independent developmental trajectories and gradually become organized into the integrated networks required for fluent mathematical reasoning [155]. Mathematical learning depends not only on the acquisition of numerical knowledge but also on the successful developmental synchronization of the diverse cognitive processes that support symbolic mathematics.

Developmental dyscalculia has traditionally been conceptualized in terms of deficits in number sense, symbolic number processing, arithmetic fact retrieval, visuospatial processing, working memory, or executive functioning [4]. Although each of these perspectives has contributed substantially to understanding mathematical learning difficulties, no single mechanism adequately explains the marked heterogeneity observed among affected children or the wide variation in developmental trajectories and educational outcomes. Increasing evidence instead suggests that mathematical competence emerges through interactions among multiple cognitive systems that gradually become integrated throughout development [23].

From the developmental timing perspective advanced in this review, dyscalculia is proposed to reflect developmental asynchrony within the systems responsible for constructing symbolic mathematical knowledge. The framework proposes that the relevant developmental challenge may involve not simply impaired numerical processing but also incomplete coordination among quantity representations, symbolic mapping, spatial organization, executive regulation, and procedural learning during periods when formal mathematics instruction increasingly assumes their functional integration. Educational difficulties may emerge when the developmental organization of these interacting systems has not yet reached the level required to support the symbolic and abstract demands of school mathematics.

The following sections examine how this developmental process unfolds. First, we consider the developmental foundations of early numerical cognition and the transition from non-symbolic quantity to symbolic number knowledge. We then examine the progressive integration of number-symbol mapping, spatial representation, and executive and procedural systems, and finally consider how developmental mismatch between these evolving neurocognitive systems and the increasing demands of mathematics education contributes to the emergence and persistence of dyscalculia.

### 5.1. Developmental Foundations of Numerical Cognition

Mathematical learning begins long before children encounter formal arithmetic instruction. During infancy and early childhood, children develop an intuitive sensitivity to quantity, magnitude, numerical order, and simple numerical relationships that provides the foundation for later symbolic mathematics [15,16,108,129]. These early numerical competencies emerge through interactions among perceptual, attentional, sensorimotor, and cognitive systems that enable children to discriminate differences in quantity, compare magnitudes, recognize small exact sets, and appreciate simple relationships such as more versus less. Although these abilities are often described collectively as early number sense, they represent a heterogeneous collection of developmental processes rather than a single unitary cognitive capacity.

Importantly, these early quantitative representations are not equivalent to formal mathematical knowledge. Infants may distinguish between larger and smaller sets or recognize approximate numerical differences without understanding number words, Arabic numerals, counting principles, or arithmetic operations [16,108]. Likewise, young children may learn to recite counting sequences before appreciating that each successive number word corresponds to an exact increase in quantity. Early numerical cognition provides an essential developmental foundation, but it must undergo substantial reorganization before it can support the symbolic and abstract forms of mathematics encountered in school.

This developmental transition requires the progressive integration of multiple representational systems. Children must learn to coordinate non-symbolic quantity representations with spoken number words, written Arabic numerals, counting sequences, spatial organization, ordinal relationships, and emerging arithmetic concepts [7,24]. These systems have different developmental origins, depend upon partially distinct neural and cognitive processes, and mature at different rates. Mathematical competence emerges not simply through the acquisition of isolated numerical skills but through the gradual functional integration of these diverse representational systems.

From a developmental timing perspective, this period represents a critical phase in which numerical representations become increasingly coordinated before the introduction of formal symbolic mathematics. Most children progressively establish stable relationships between quantities, number words, symbols, and counting procedures through repeated interactions with their physical and educational environments [7]. However, developmental trajectories vary considerably across individuals. Differences in the rate at which these representational systems become integrated may influence children’s readiness to acquire more complex mathematical concepts once formal instruction begins.

Importantly, developmental asynchrony during this stage should not be interpreted as evidence of a generalized impairment in mathematical ability. Individual component systems may develop relatively normally while remaining insufficiently coordinated to support fluent symbolic learning. A child may demonstrate appropriate intuitive sensitivity to quantity yet struggle to connect these representations with verbal labels or written numerals. Conversely, another child may learn counting routines accurately while showing only limited understanding of the quantitative relationships that those routines represent. These patterns illustrate that successful mathematical development depends not only on the maturation of individual cognitive processes but also on their progressive integration into coherent representational networks.

This developmental framework provides the foundation for conceptualizing dyscalculia as potentially involving differences in developmental coordination rather than solely a deficit in number sense or arithmetic ability. The following section examines how the transition from intuitive numerical representations to formal symbolic mathematics depends upon the successful development of number-symbol mapping and why this process represents a critical point of vulnerability in the emergence of mathematical learning difficulties.

### 5.2. Number-Symbol Mapping and Mathematical Representation

The transition from intuitive numerical understanding to formal mathematics depends upon the successful coordination of multiple representational systems. Children must learn that quantities, spoken number words, written Arabic numerals, ordinal sequences, arithmetic symbols, and mathematical procedures all refer to related aspects of the same numerical concepts. This developmental process extends well beyond learning numerical labels or memorizing counting routines. Rather, it involves constructing increasingly stable relationships among diverse representations that together support flexible mathematical reasoning [7,24].

Number-symbol mapping represents a critical stage in this developmental progression. Within the present framework, the developmental coordination of these representations, rather than mastery of any single representation alone, constitutes the primary developmental construct. A child may possess an intuitive understanding that one collection contains more objects than another while experiencing considerable difficulty linking that quantity to the corresponding spoken number word or written numeral [24]. Conversely, children may accurately recite counting sequences or recognize Arabic numerals without fully understanding the quantitative relationships they represent [7]. Symbolic mathematical knowledge develops through the progressive integration of quantitative meaning with linguistic, visual, and procedural representations rather than through the acquisition of isolated numerical facts.

Successful number-symbol integration also requires children to coordinate multiple dimensions of numerical knowledge simultaneously. The written symbol “7,” the spoken word “seven,” the corresponding quantity, its ordinal position within the counting sequence, its relative magnitude compared with other numbers, and its role within arithmetic operations must gradually become integrated into a coherent representational network [108,116,129,147]. As these associations strengthen through development, children become increasingly able to move flexibly between concrete quantities, symbolic notation, verbal reasoning, and mathematical procedures without relying on laborious intermediate strategies.

From a developmental timing perspective, this integration is neither instantaneous nor uniform. Different representational systems mature at different rates, and their successful coordination depends upon repeated interactions across home, educational, and everyday numerical experiences. For many children, these mappings become progressively automatized through counting, comparison, estimation, and arithmetic practice [7]. For others, however, these representational systems remain only partially integrated during the period when formal mathematics instruction increasingly assumes fluent symbolic understanding. Educational difficulties may arise because instruction advances more rapidly than the developmental organization of the underlying representational network.

This developmental interpretation extends contemporary models emphasizing the approximate number system, symbolic number processing, and number-magnitude mapping [24]. Rather than viewing these mechanisms as competing explanations, they may be understood as describing complementary components of a broader developmental process through which intuitive numerical representations gradually become integrated with symbolic mathematical knowledge. Dyscalculia need not arise from a single impaired mechanism. Instead, difficulties may emerge whenever the developmental coordination of quantity representations, numerical symbols, language, and procedural knowledge remains incomplete during periods of increasing educational demand.

The consequences of incomplete number-symbol integration extend throughout mathematical learning. Children may demonstrate slow numerical comparison, inefficient magnitude estimation, immature counting strategies, weak arithmetic fact retrieval, or difficulty generalizing mathematical concepts across different representational formats [4,24]. Importantly, these diverse manifestations may reflect, among other possible pathways, the incomplete functional integration of representational systems that normally support fluent symbolic mathematics. Mathematical performance consequently becomes slow, effortful, and vulnerable to increasing complexity because fundamental numerical representations have not yet become sufficiently coordinated to support automatic processing.

Viewed within this developmental framework, number-symbol mapping represents far more than an early instructional milestone. It is the process through which intuitive quantitative knowledge is transformed into the flexible symbolic representations that support arithmetic, mathematical reasoning, and later abstract thought. The following section considers how this emerging representational system becomes further shaped by spatial organization, executive control, procedural learning, and working memory as mathematics becomes increasingly complex.

### 5.3. Spatial, Executive, and Procedural Difficulties in Development

As children’s mathematical understanding becomes increasingly symbolic, successful performance depends on the progressive integration of numerical representations with spatial cognition, executive control, working memory, and procedural learning. These systems do not simply support mathematical performance independently; rather, they interact continuously to organize increasingly complex numerical reasoning. Consequently, mathematics develops through the coordinated maturation of multiple cognitive systems whose developmental trajectories only gradually converge during childhood.

Spatial representations play a particularly important role in this process. Numerical magnitude is frequently organized spatially, allowing children to compare quantities, understand numerical order, estimate relative magnitude, and appreciate relationships among numbers. The mental number line has often been proposed as a framework for understanding this spatial organization, although contemporary research suggests that its development is gradual, heterogeneous, and influenced by both experience and educational instruction rather than representing a fixed cognitive structure [38,44,54]. From a developmental perspective, spatial representations become increasingly calibrated as children learn to coordinate quantity, symbolic notation, ordinal relationships, and spatial position within increasingly abstract mathematical tasks.

These developmental demands become particularly evident during the acquisition of place-value concepts. Understanding multidigit numbers requires children to integrate numerical magnitude with positional notation, base-ten organization, language, and symbolic representation [4]. The meaning of an individual digit changes according to its position within the numeral, requiring children to inhibit purely perceptual interpretations while simultaneously coordinating quantity, spatial organization, and symbolic rules. Place-value understanding illustrates how mathematical competence depends upon the successful integration of multiple representational systems rather than on procedural knowledge alone. Children who acquire computational procedures without fully integrating these underlying representations may perform algorithms accurately in familiar situations while experiencing substantial difficulty transferring their knowledge to novel mathematical problems.

Executive functions contribute an additional layer of developmental organization. Their contribution depends not only on their individual maturity but also on the extent to which they become coordinated with emerging numerical representations during development. Mathematical problem-solving requires children to maintain intermediate results in working memory, inhibit ineffective solution strategies, shift flexibly between procedures, monitor ongoing performance, and organize sequential operations toward a desired outcome [9,140,147]. These executive processes become increasingly important as mathematical tasks progress from simple counting toward multidigit calculation, algebraic reasoning, and complex problem-solving. Rather than serving as separate cognitive resources, executive functions interact continuously with numerical representations, enabling children to coordinate symbolic knowledge with procedural execution.

Working memory is especially important because mathematics frequently requires information to be maintained while successive computational operations are performed. Intermediate values must remain available long enough to guide subsequent calculations, relationships among quantities must be preserved throughout multistep procedures, and errors must be detected before they accumulate [127]. When foundational number-symbol mappings remain inefficient, working-memory demands increase substantially because children must devote cognitive resources to interpreting numerical representations that have not yet become automatic. Fewer resources remain available for higher-order reasoning, strategic planning, and conceptual understanding, contributing to the effortful and inconsistent performance frequently observed in children with dyscalculia.

Procedural learning likewise develops through repeated coordination among numerical representations, executive regulation, memory, and strategy selection. Children typically progress from manipulating concrete objects, to verbal counting, to derived computational strategies, and eventually to rapid retrieval of arithmetic facts and flexible problem-solving [7]. This developmental progression reflects increasing functional integration rather than simple memorization. Automatization emerges as frequently coordinated numerical relationships become progressively consolidated, reducing the cognitive resources required for routine calculations and allowing attention to shift toward more complex mathematical reasoning.

From a developmental timing perspective, difficulties in dyscalculia may arise when these supporting systems mature asynchronously. Number representations may be relatively well established while executive regulation, spatial organization, or procedural learning remain less fully integrated. Alternatively, executive and working-memory capacities may develop appropriately while symbolic numerical representations remain unstable. These differing developmental trajectories help explain the marked heterogeneity of dyscalculia, in which children may present with distinct cognitive profiles despite experiencing similar academic difficulties [4,23]. Rather than reflecting multiple unrelated disorders, these profiles may represent different patterns of developmental asynchrony within the broader neurocognitive network supporting mathematical learning.

Viewed collectively, spatial cognition, executive control, working memory, and procedural learning illustrate that mathematical competence depends upon far more than numerical knowledge alone. Successful mathematics emerges through the progressive functional integration of these interacting systems as symbolic representations become increasingly abstract and educational demands continue to expand. The following section examines how developmental differences within these interacting systems become increasingly evident during formal mathematics education, contributing to educational mismatch, developmental cascades, and the heterogeneous presentation of dyscalculia.

### 5.4. From Concrete Quantity to Abstract Mathematics: Developmental Mismatch and Cascades

A developmental timing framework helps explain why dyscalculia frequently becomes more apparent as mathematics instruction progresses from concrete numerical experiences to increasingly abstract symbolic reasoning. During early childhood, many numerical activities are supported by physical objects, visual representations, finger counting, and direct manipulation of quantities. These concrete experiences reduce reliance on fully internalized symbolic representations by allowing children to see, touch, compare, and manipulate numerical relationships directly [7]. As a result, early numerical competence may appear relatively intact even when the developmental integration of quantity, symbol, language, and spatial representation remains incomplete.

Formal mathematics education progressively alters these developmental demands. Children are increasingly expected to operate on written numerals rather than concrete objects, understand place value, retrieve arithmetic facts efficiently, solve multistep calculations, and manipulate abstract mathematical relationships without external supports [15,38,49,130]. These transitions require that quantity representations, symbolic notation, spatial organization, executive control, working memory, and procedural knowledge have become sufficiently integrated to support increasingly automatic mathematical processing. When these interacting systems remain developmentally asynchronous, a growing mismatch may emerge between the child’s current neurocognitive organization and the symbolic demands imposed by the mathematics curriculum [97,107,142,147].

From this perspective, dyscalculia does not suddenly appear when mathematical content becomes more difficult. Rather, the progression of formal instruction exposes developmental differences that may have had relatively little functional consequence during earlier stages of learning. Educational difficulties may become increasingly apparent because school mathematics places progressively greater demands on internally coordinated numerical representations while providing fewer perceptual and motor supports. Children who previously relied on concrete strategies may experience increasing difficulty as instruction assumes the automatic integration of numerical meaning, symbolic notation, procedural knowledge, and abstract reasoning.

These developmental mismatches can initiate cascading effects throughout mathematical learning. Early difficulties linking quantities to symbolic representations may reduce the efficiency of arithmetic practice, limiting opportunities to consolidate numerical facts, refine computational strategies, and develop procedural fluency [24]. Because later mathematical concepts are cumulative, weaknesses in foundational numerical representations may gradually influence increasingly complex domains, including multidigit calculation, fractions, algebraic thinking, mathematical reasoning, and problem-solving [4]. Consequently, relatively modest developmental differences during early number learning may broaden into more extensive mathematical difficulties through cumulative interactions between learning and development.

The consequences of these cascades extend beyond mathematical performance alone. Repeated experiences of unsuccessful problem-solving, slow progress, and increasing instructional difficulty may contribute to reduced mathematical confidence, avoidance of challenging tasks, lower academic self-efficacy, and the development of mathematics anxiety [6,86,119]. These secondary consequences may further reduce engagement with mathematical learning, limiting practice opportunities precisely when continued experience is needed to strengthen emerging numerical representations. Thus, developmental differences in early numerical integration may become progressively amplified through reciprocal interactions between the learner and the educational environment.

Importantly, a developmental timing framework emphasizes that these developmental cascades are neither inevitable nor irreversible. Numerical representations, executive functions, and procedural knowledge continue to mature throughout childhood, while educational experiences remain capable of shaping their functional integration. Interventions that strengthen connections among quantity, language, symbolic notation, spatial representation, and procedural understanding may help reduce developmental mismatch by supporting the progressive consolidation of foundational numerical concepts rather than emphasizing procedural fluency alone [4]. Such approaches are consistent with evidence that meaningful conceptual understanding provides a more stable foundation for later mathematical learning than rote procedural instruction in isolation.

Viewed from this developmental perspective, dyscalculia illustrates the reciprocal relationship between neurodevelopment and mathematics education. Academic success depends not only on children’s numerical abilities but also on whether the developmental organization of the multiple systems supporting symbolic mathematics has progressed sufficiently to meet the increasing cognitive demands of formal instruction. When educational expectations outpace this developing functional integration, the framework proposes that mathematical learning becomes increasingly vulnerable to developmental mismatch and cascading educational consequences. Conversely, educational environments that provide appropriate developmental scaffolding can strengthen these emerging representational networks and promote more successful mathematical learning trajectories.

### 5.5. Synthesis: Dyscalculia as a Developmental Timing Disorder

The developmental timing framework advanced in this review does not replace established theories of dyscalculia but provides a broader developmental context within which they can be understood. Models emphasizing deficits in number sense, symbolic number processing, number-magnitude mapping, visuospatial representation, working memory, executive functioning, or procedural learning each identify important contributors to mathematical development. Rather than representing competing explanations, these perspectives describe interacting components of a developing mathematical system whose successful coordination is necessary for fluent numerical reasoning. Their apparent diversity reflects the complexity of mathematical cognition rather than the existence of mutually exclusive causal mechanisms [33,97,121,140,147].

From this perspective, dyscalculia is proposed to reflect a disorder of developmental organization within distributed neurocognitive systems supporting symbolic mathematics. Numerical competence depends on the progressive integration of quantity representations, language, symbolic notation, spatial organization, executive control, working memory, procedural learning, and abstraction. Each of these systems contributes to mathematical performance because it operates as part of an increasingly integrated developmental network. Within the framework, mathematical learning is proposed to become more vulnerable when these interacting systems mature along partially independent trajectories and have not achieved sufficient functional coordination to support the symbolic and procedural demands of formal mathematics instruction [57,97,140,155].

Importantly, developmental asynchrony should not be interpreted as evidence of a simple developmental delay or a generalized weakness in mathematical ability. Individual component systems may develop appropriately while remaining insufficiently integrated to support efficient mathematical reasoning. Consequently, children with dyscalculia often present with markedly different cognitive profiles despite experiencing similar educational difficulties. Some may demonstrate relatively preserved intuitive quantity processing but struggle with symbolic mapping, whereas others may experience greater difficulty with executive regulation, spatial organization, arithmetic fluency, or procedural learning. This heterogeneity is consistent with a developmental systems perspective in which different patterns of maturational asynchrony may contribute to different pathways toward comparable educational outcomes [4,23].

The developmental timing framework also emphasizes the reciprocal relationship between neurodevelopment and mathematics education. Mathematical understanding develops through continuous interactions between maturing neurocognitive systems and increasingly sophisticated educational experiences. Instruction not only builds mathematical knowledge but also shapes the functional organization of the cognitive systems that support numerical reasoning. The developmental timing framework also generates specific empirical predictions. Longitudinal studies should demonstrate that measures of developmental coordination among quantity representation, symbolic number knowledge, spatial organization, executive control, and procedural learning predict later mathematical achievement more accurately than isolated measures of any single component. Likewise, interventions introduced during periods of emerging coordination among these interacting systems should produce broader and more durable improvements than interventions based solely on chronological age, diagnostic category, or current mathematical performance. Confirmation of these predictions would provide evidence supporting the hypothesis that cross-system developmental coordination contributes independently to mathematical learning.

Educational environments that provide developmentally appropriate scaffolding, strengthen conceptual understanding, promote meaningful connections among multiple numerical representations, and progressively reduce external supports may facilitate the integration of these developing systems while limiting the accumulation of secondary academic difficulties [4].

Dyscalculia illustrates the same developmental principle identified in dyslexia and ADHD despite involving different neurocognitive networks. In dyslexia, educational difficulties may emerge primarily from developmental variation in the integration of language and print. In ADHD, they are proposed to relate principally to developmental variation in executive and regulatory organization. In dyscalculia, they are proposed to relate to developmental variation in the integration of numerical representations with symbolic, spatial, executive, and procedural systems. Although the specific cognitive mechanisms differ, each disorder is proposed to illustrate the same fundamental developmental principle: learning difficulties may become more likely when increasing educational demands exceed the current level of functional integration achieved by the underlying neurodevelopmental systems.

The preceding sections suggest that dyslexia, ADHD, and dyscalculia should not be viewed simply as isolated disorders affecting reading, attention, or mathematics. Rather, within the present framework, they are conceptualized as different expressions of a common developmental principle in which learning depends upon the progressive coordination of multiple interacting neurocognitive systems across time. This broader perspective provides the foundation for the general developmental timing theory presented in the following discussion, where the common mechanisms linking diverse learning disabilities are considered within a unified framework of neurodevelopment, education, and developmental systems organization.

## 6. Mixed Learning Profiles and the Limits of Domain-Specific Labels

The preceding sections have examined dyslexia, ADHD, and dyscalculia as distinct learning disabilities, each characterized by its own developmental trajectory, neurocognitive mechanisms, and educational manifestations. This disorder-specific approach remains clinically valuable because it facilitates diagnosis, communication, educational planning, and intervention. However, increasing evidence suggests that these diagnostic categories often fail to capture the complexity of children’s developmental profiles. Many learners experience difficulties that extend across traditional diagnostic boundaries, reflecting interactions among multiple cognitive systems rather than isolated impairments confined to a single academic domain [1,2].

The high rates of co-occurrence observed among reading, attention, language, and mathematical difficulties have prompted growing interest in transdiagnostic and multiple-deficit models of neurodevelopmental disorders [69,87,127]. These perspectives emphasize that cognitive systems such as executive function, working memory, processing speed, language, attention, and motor control contribute to learning across multiple academic domains. Consequently, developmental variation within one of these systems may influence reading, mathematics, written expression, classroom behavior, and other aspects of learning simultaneously. Mixed learning profiles should be expected rather than regarded as unusual exceptions.

The developmental timing framework proposed in this review extends these perspectives by emphasizing not only which neurocognitive systems contribute to learning but also how their developmental coordination changes over time. The framework proposes that academic difficulties may become more likely when interacting systems have not achieved sufficient functional integration to meet the increasing cognitive demands of formal education. Because many of these systems support more than one area of learning, maturational asynchrony within a single component system may contribute to difficulties across multiple academic domains, while different patterns of developmental asynchrony may contribute to superficially similar educational outcomes. Within the present framework, mixed learning profiles are conceptualized as potentially reflecting shared neurocognitive architecture rather than simply the co-occurrence of independent disorders.

The following sections examine the implications of this perspective. We first consider why domain-specific diagnostic categories, although clinically useful, provide only a partial account of children’s developmental organization. We then examine the shared neurocognitive systems that contribute across learning disorders, explore how developmental asynchrony gives rise to mixed learning profiles, and consider the implications of this transdiagnostic perspective for assessment, educational intervention, and developmental theory.

### 6.1. Beyond Single-Domain Disorders

Learning disabilities have traditionally been classified according to the academic domain in which difficulties are most apparent. Dyslexia is identified primarily through persistent problems in reading and written language, ADHD through impairments in attention, behavioral regulation, and executive functioning, and dyscalculia through difficulties in numerical cognition and mathematical learning. These diagnostic distinctions have considerable clinical value because they facilitate assessment, communication among professionals, educational planning, and the development of targeted interventions [1,2]. Domain-specific classifications remain an essential component of educational and clinical practice.

Despite their practical usefulness, however, these categories do not fully reflect the developmental organization of learning. Reading, mathematics, attention, written expression, and classroom behavior do not emerge from isolated neurocognitive modules that mature independently. Instead, they develop through interactions among multiple cognitive, linguistic, perceptual, motor, and executive systems that gradually become coordinated throughout childhood [57,87,140]. Consequently, the boundaries separating individual learning disabilities are often less distinct at the level of underlying developmental mechanisms than they appear from their academic manifestations.

This distinction between clinical description and developmental explanation is important. Diagnostic labels describe where learning difficulties become most visible, but they do not necessarily explain how those difficulties arise. A diagnosis of dyslexia identifies persistent difficulty acquiring fluent reading, yet it does not specify the developmental interactions among phonological processing, language, visual recognition, executive regulation, and learning experience that may have contributed to that outcome. Similarly, diagnoses of ADHD and dyscalculia characterize characteristic behavioral or academic profiles without fully explaining how interactions among multiple developing neurocognitive systems give rise to those patterns over time.

From a developmental timing perspective, diagnostic categories may be viewed as descriptions of predominant educational outcomes rather than as boundaries separating fundamentally distinct developmental processes. Different learning disabilities involve different neurocognitive systems and different developmental pathways, but they also depend upon many of the same underlying processes, including attention, working memory, executive regulation, language, processing speed, motor organization, and symbolic learning. Variation in the developmental organization of these shared systems may influence more than one academic domain simultaneously, making overlap among learning disabilities potentially understandable as a consequence of shared developmental architecture rather than solely as evidence of unrelated disorders occurring together by chance. This perspective does not diminish the importance of domain-specific diagnoses. Rather, it complements them by shifting attention toward the interacting developmental systems that underlie academic performance. Understanding how these systems mature, coordinate, and respond to increasing educational demands provides a broader framework for explaining why children frequently present with mixed learning profiles and why similar developmental vulnerabilities may be expressed differently across individuals and across stages of schooling.

The following section examines these shared developmental systems in greater detail and considers how their interactions contribute to learning across traditional diagnostic boundaries.

### 6.2. Shared Developmental Systems Across Learning Disorders

The overlap among learning disabilities becomes more understandable when considered from the perspective of shared developmental systems rather than isolated academic domains. Reading, mathematical reasoning, written expression, attention, and classroom self-regulation all depend upon a relatively small number of neurocognitive systems that contribute across multiple forms of learning. These systems do not operate independently but develop through continuous interactions that gradually become coordinated throughout childhood. Consequently, developmental variation within one component system may influence performance across several academic domains simultaneously, providing a developmental explanation for the frequent overlap observed among learning disabilities [1].

Processing speed represents one such shared system. Efficient learning depends upon the rapid coordination of perception, cognitive processing, and motor responses across a wide range of educational activities. Processing speed contributes to fluent word recognition, arithmetic fact retrieval, written production, visual scanning, classroom participation, and the efficient completion of academic tasks [3]. Developmental variation in processing efficiency may influence reading, mathematics, and written language simultaneously, even though difficulties become most apparent within one academic domain.

Working memory similarly supports learning across diagnostic categories. Children must temporarily maintain and manipulate information while decoding text, comprehending sentences, solving multistep mathematical problems, organizing written expression, following classroom instructions, and regulating goal-directed behavior [69,97,127]. Because working memory contributes to so many aspects of learning, developmental differences in its maturation may contribute to difficulties that extend well beyond any single academic subject.

Executive regulation provides another common developmental foundation. Planning, inhibitory control, cognitive flexibility, error monitoring, and self-regulation enable children to organize behavior across increasingly complex learning situations [16,66,143]. These processes support successful reading comprehension, mathematical problem-solving, written composition, classroom participation, and independent learning. Their contribution extends across traditional diagnostic boundaries, influencing both academic achievement and the self-regulatory capacities required for effective classroom learning.

Language also functions as a transdiagnostic developmental system. Although language is most obviously associated with reading development, it also supports mathematical vocabulary, verbal reasoning, self-directed speech, problem-solving, comprehension of classroom instruction, and social communication [4]. Likewise, attentional control contributes to sustained practice, error detection, strategy selection, and the consolidation of newly acquired skills across reading, mathematics, and written expression [16,66,143]. These systems illustrate that cognitive processes typically assigned to one learning disability often contribute broadly to many aspects of academic development.

Motor organization and temporal coordination provide further examples of shared developmental architecture. Fine motor control, eye-movement coordination, handwriting, response inhibition, rhythmic timing, and the temporal sequencing of actions support reading fluency, written production, arithmetic procedures, and classroom behavior [56,150]. These motor and timing processes are not ancillary to learning but contribute directly to the organization and automatization of increasingly complex academic skills throughout development.

Finally, symbolic learning itself represents a common developmental challenge that extends across multiple academic domains. Learning requires children to construct increasingly stable relationships between symbols and their underlying meanings, whether linking letters with speech sounds, Arabic numerals with numerical magnitude, written words with semantic concepts, or visual notation with procedural rules. Although the specific symbols differ across reading, mathematics, and written language, the developmental process of establishing and consolidating symbolic representations follows similar principles of progressive integration and increasing automaticity [40,116,129]. Successful academic learning depends not only on acquiring individual symbolic systems but also on coordinating those systems with broader cognitive, linguistic, executive, and perceptual processes.

Viewed collectively, these shared developmental systems illustrate why learning disabilities rarely conform to sharply defined academic boundaries. Reading, attention, mathematics, written language, and classroom behavior emerge from overlapping neurocognitive networks that mature along partially independent developmental trajectories while simultaneously becoming increasingly integrated. The developmental timing framework predicts that variation within any one of these foundational systems may influence multiple areas of learning, producing the mixed developmental profiles commonly observed in educational practice. The following section examines how these shared developmental systems give rise to overlapping patterns of learning difficulty and why mixed learning profiles should be regarded as an expected consequence of neurodevelopment rather than an exception to it.

### 6.3. Developmental Asynchrony and Mixed Learning Profiles

Mixed learning profiles are often described in terms of comorbidity, implying that two or more independent disorders occur simultaneously within the same child. Although this description is clinically convenient, it may not adequately capture the developmental processes that give rise to overlapping patterns of learning difficulty. From a developmental timing perspective, mixed learning profiles are proposed to reflect interactions among neurocognitive systems that mature at different rates and contribute to multiple domains of learning simultaneously [1,57,87,127]. Because many foundational systems support more than one academic function, developmental asynchrony within a single system may contribute to difficulties that extend across traditional diagnostic boundaries.

This perspective helps explain why children rarely present with neatly circumscribed learning difficulties. A child referred because of poor reading may also experience weaknesses in attention, working memory, handwriting, processing speed, oral language, or mathematics [1]. Similarly, a child diagnosed with ADHD may demonstrate reduced reading fluency, written expression, or mathematical achievement, while a child with dyscalculia may also experience difficulties involving language, executive regulation, visuospatial processing, or attention [4,69,87,121]. These combinations should not necessarily be interpreted as multiple unrelated disorders occurring together. Rather, they may reflect the contribution of shared developmental systems to several forms of academic learning at the same time.

The developmental pathways leading to these mixed profiles may differ substantially across children. For example, reduced attentional stability and executive regulation in ADHD may limit opportunities for sustained reading practice, slowing orthographic learning and the development of reading fluency despite relatively preserved phonological skills [63,143]. Conversely, persistent phonological or language weaknesses associated with dyslexia may influence mathematical learning when tasks depend heavily on number-word knowledge, verbal working memory, or symbolic language [87,97]. Likewise, children with dyscalculia may appear inattentive or poorly organized during mathematics instruction because complex calculations impose substantial demands on working memory, procedural sequencing, error monitoring, and emotional regulation rather than because of primary deficits in attentional control [4,97,107,119]. In each instance, the framework proposes that the observable academic profile may reflect interactions among multiple developing systems rather than dysfunction within a single isolated domain.

This developmental interpretation also helps explain why the expression of learning difficulties often changes across development. As educational demands evolve, the relative contribution of different neurocognitive systems also changes. Early phonological weaknesses may become increasingly apparent during reading instruction, subtle executive-regulatory differences may emerge during periods requiring greater independence and sustained attention, and initially modest weaknesses in number-symbol integration may become more evident as mathematics shifts toward increasingly abstract symbolic reasoning [24,121,148]. Consequently, the developmental profile observed at one stage of schooling may differ substantially from that seen several years later, even though the underlying neurodevelopmental architecture has remained broadly continuous.

Importantly, developmental asynchrony does not imply that every child with one learning disability will inevitably develop additional academic difficulties. Rather, it suggests that the expression of vulnerability depends upon the interaction between the child’s evolving neurocognitive organization and the specific cognitive demands imposed by the educational environment. Protective factors, compensatory strategies, instructional quality, family support, and individual strengths may all influence whether developmental differences remain circumscribed or broaden into more extensive learning profiles [33,57,87]. Mixed learning profiles represent one possible developmental outcome rather than an inevitable consequence of early neurodevelopmental variation.

Viewed collectively, these observations suggest that overlap among learning disabilities may be understood as an expected feature of human neurodevelopment rather than an anomaly requiring multiple independent explanations. Reading, mathematics, attention, language, executive regulation, and motor organization are supported by overlapping developmental systems whose trajectories remain partially independent while becoming progressively integrated throughout childhood. Different patterns of developmental asynchrony within these shared systems may contribute to diverse combinations of academic strengths and weaknesses while reflecting common developmental principles. This perspective shifts the emphasis from explaining why disorders co-occur to examining how interactions between developing systems may contribute to the heterogeneous learning profiles commonly encountered in educational and clinical practice.

The following section considers the practical implications of this developmental systems perspective for educational assessment and intervention, with particular emphasis on why transdiagnostic approaches may better address children’s underlying developmental needs than interventions directed exclusively toward individual diagnostic categories.

### 6.4. Educational Consequences and Transdiagnostic Intervention

Recognizing that learning disabilities may reflect interactions among developmental systems has important implications for educational assessment and intervention. Traditional approaches often begin by identifying the academic domain in which a child experiences the greatest difficulty and then selecting interventions targeted specifically toward that domain. This strategy remains valuable because reading, mathematics, and attentional difficulties each require specialized instructional approaches. However, when learning difficulties reflect developmental asynchrony across multiple interacting systems, interventions directed exclusively toward a single academic symptom may not fully address the underlying sources of educational difficulty [2].

A developmental timing framework encourages assessment that extends beyond academic performance to examine the neurocognitive systems supporting learning. Difficulties in reading, mathematics, or classroom behavior should be interpreted within the broader context of language development, executive regulation, working memory, processing speed, attentional control, motor organization, symbolic learning, and developmental history. Such an approach does not replace conventional diagnostic assessment but complements it by identifying the interacting developmental processes that contribute to a child’s educational profile. Consequently, assessment becomes concerned not only with what a child finds difficult but also with why those difficulties have emerged and which developmental systems require further support.

This perspective also has important implications for intervention. Reading intervention may produce limited progress when attentional regulation, rapid naming, oral language, or working memory continue to constrain the acquisition of fluent decoding and orthographic knowledge [121]. Likewise, mathematics intervention may be less effective if difficulties involving spatial representation, executive sequencing, language, or working memory remain unaddressed [4]. Similarly, interventions targeting attentional regulation may improve classroom participation without automatically producing gains in reading fluency or mathematical reasoning unless opportunities for repeated academic practice and skill consolidation are incorporated into instruction [62,153]. Effective intervention depends upon understanding how multiple developmental systems interact to support learning rather than focusing exclusively on the most visible academic symptom.

A developmental systems perspective also encourages greater flexibility in educational planning. Children who receive the same diagnostic label may require substantially different intervention priorities because the developmental pathways underlying their learning difficulties differ. Conversely, children carrying different diagnoses may benefit from overlapping educational supports when they share vulnerabilities in executive regulation, language, processing speed, working memory, or symbolic learning. Assessment and intervention should be guided by individual developmental profiles rather than by diagnostic categories alone, recognizing that similar educational outcomes may arise through different developmental pathways and that similar developmental vulnerabilities may influence multiple academic domains.

This perspective is consistent with contemporary transdiagnostic approaches that emphasize dimensions of cognitive functioning across diagnostic boundaries while retaining the practical value of established diagnostic classifications [1]. The developmental timing framework extends these approaches by emphasizing that intervention should consider not only the current organization of cognitive systems but also their developmental trajectory. Educational planning becomes a process of supporting the progressive coordination of developing neurocognitive systems while ensuring that instructional demands remain appropriately aligned with the child’s evolving developmental readiness.

Table 1 summarizes the principal developmental timing vulnerabilities proposed across dyslexia, ADHD, dyscalculia, and mixed learning profiles. It links each characteristic academic presentation with the principal neurocognitive systems involved the proposed developmental asynchrony, the likely developmental cascade, and the corresponding educational implications. The developmental timing framework generates several predictions that distinguish it from traditional comorbidity models. Longitudinal studies should demonstrate that measures of developmental coordination among shared neurocognitive systems predict the emergence of mixed learning profiles more accurately than the presence or severity of isolated deficits within individual domains. Likewise, interventions targeting shared developmental systems during periods of emerging cross-system coordination should reduce the likelihood of cross-domain learning difficulties more effectively than interventions directed exclusively toward single academic symptoms. These predictions provide a direct empirical basis for evaluating the developmental timing account against conventional disorder-specific models.

**Figure 2:**
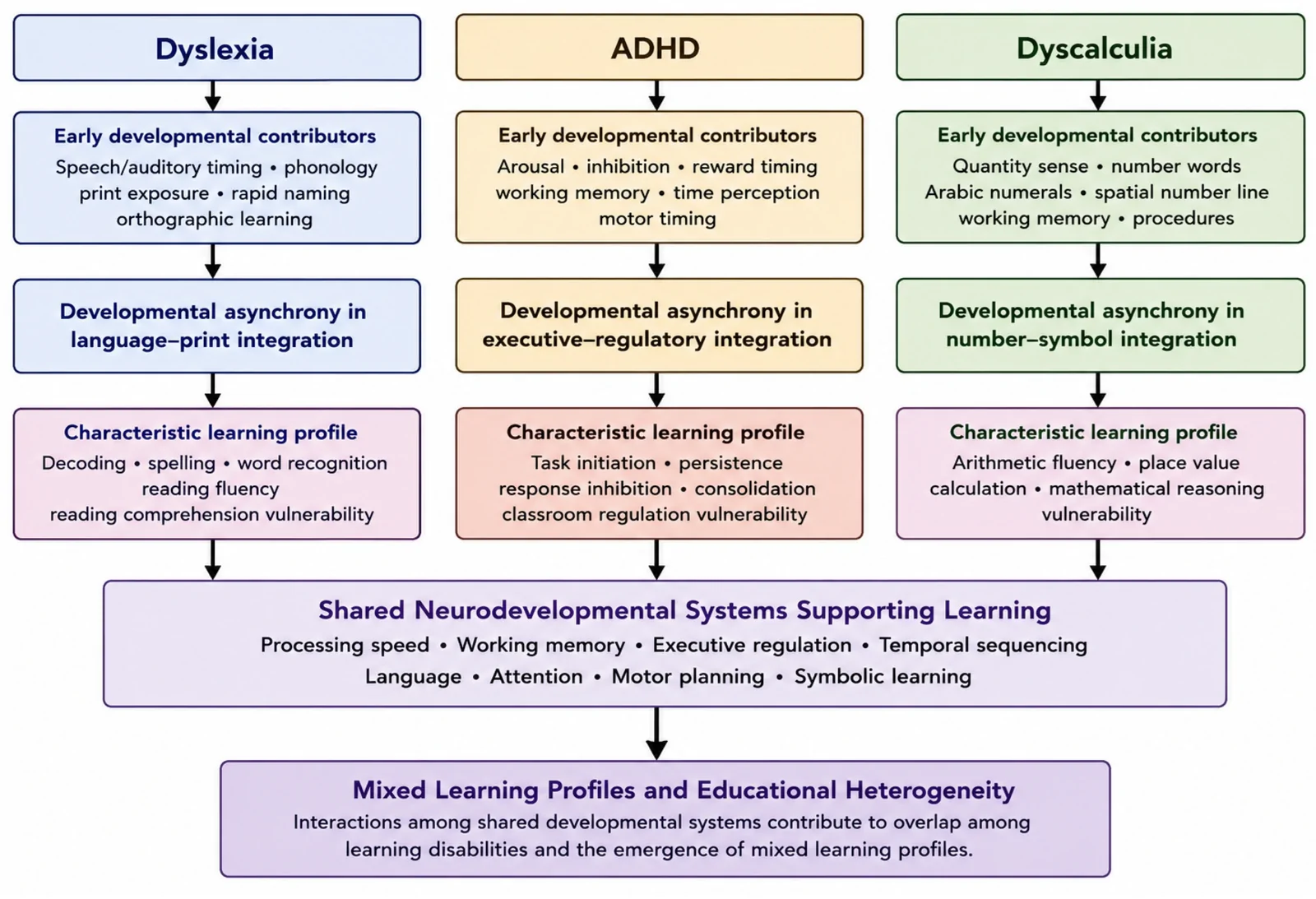
Shared developmental systems underlying dyslexia, ADHD, dyscalculia, and mixed learning profiles.

Figure 2 illustrates how dyslexia, ADHD, and dyscalculia differ in their predominant academic manifestations while sharing multiple developmental systems, including language, executive regulation, working memory, processing speed, attention, motor organization, temporal coordination, and symbolic learning. Developmental asynchrony within these shared systems may contribute to different patterns of learning difficulty and helps explain the high frequency of mixed learning profiles. The model emphasizes that diagnostic categories describe the principal educational manifestation, whereas underlying developmental systems extend across traditional disorder boundaries.

### 6.5. Synthesis: Toward a Developmental Systems Model of Learning Disabilities

The developmental timing framework proposed in this review extends beyond explaining individual learning disabilities to provide a broader account of why diverse learning profiles frequently overlap throughout development. Dyslexia, ADHD, and dyscalculia remain clinically meaningful diagnostic categories because they describe the predominant academic or behavioral manifestations of learning difficulties. However, within the present framework, they are not regarded as entirely independent developmental entities. Rather, they are conceptualized as different expressions of interacting neurocognitive systems whose developmental trajectories only partially coincide and whose successful coordination is required for adaptive learning.

From this perspective, the framework proposes that learning disabilities may involve differences in functional integration rather than solely isolated deficits confined to specific academic domains. Reading, mathematical reasoning, attention, executive regulation, language, processing speed, motor organization, working memory, and symbolic learning each develop along partially independent trajectories while simultaneously becoming integrated through ongoing interactions among brain maturation, experience, and education. The framework proposes that more effective developmental coordination should be associated with increasingly fluent and flexible academic skills. In contrast, incomplete functional integration during periods of escalating educational demand may increase vulnerability to learning difficulties in ways that reflect the particular developmental systems involved.

Importantly, this perspective provides a potential account of both the diversity and the heterogeneity of learning disabilities. Different children may present with similar academic difficulties despite following different developmental pathways. In contrast, children with similar developmental vulnerabilities may exhibit markedly different educational profiles depending upon which neurocognitive systems are most affected, the timing of developmental divergence, and the educational contexts in which those vulnerabilities become expressed. Consequently, within the present framework, variability and overlap are viewed as potentially expected features of complex developmental systems operating under changing environmental demands rather than necessarily as anomalies requiring multiple independent explanations.

The developmental timing framework also provides a conceptual bridge between traditional diagnostic approaches and contemporary transdiagnostic models of neurodevelopment. Diagnostic categories remain valuable for clinical communication, educational planning, and research, but they become more informative when interpreted within the broader context of interacting developmental systems. This perspective encourages clinicians, educators, and researchers to move beyond asking which diagnostic category best characterizes a child’s learning difficulties and instead consider how multiple neurocognitive systems have developed, how they currently interact, and which aspects of their functional coordination require further support.

More broadly, this developmental systems perspective conceptualizes learning disabilities as dynamic developmental phenomena rather than fixed neurocognitive conditions. Development continues throughout childhood and adolescence, educational experiences remain capable of modifying developmental trajectories, and neurocognitive systems continue to reorganize in response to instruction, practice, and environmental support. Consequently, learning profiles should be expected to evolve as interactions among biological maturation, educational experience, and environmental opportunity reshape the functional organization of cognition. A developmental timing framework emphasizes trajectories of development rather than static diagnostic states. Diagnostic categories are interpreted within the present framework as useful descriptions of predominant developmental outcomes rather than as discrete developmental mechanisms.

The preceding sections suggest that, within the proposed framework, the traditional distinction among dyslexia, ADHD, dyscalculia, and mixed learning profiles may reflect differences in the predominant expression of developmental asynchrony rather than fundamentally different developmental principles. Each disorder is proposed to highlight a different expression of a broader developmental process involving the progressive coordination of interacting neurocognitive systems that support learning. This shared developmental architecture provides the foundation for the general developmental timing theory developed in the following discussion, where the broader implications for neurodevelopment, education, developmental systems theory, and future research are considered.

## 7. Discussion: What a Developmental Timing Account Adds

The developmental timing account advanced in this review does not replace established explanations of dyslexia, ADHD, or dyscalculia. Phonological processing remains central to dyslexia, executive and motivational regulation to ADHD, and number-symbol mapping to dyscalculia [16,19,24]. Its principal contribution is to reorganize these established mechanisms within a broader developmental architecture in which developmental timing, cross-system coordination, and the alignment between maturation and educational demand are proposed as additional explanatory variables [10,11].

The central question is not only which component system is weak, but whether the systems required for academic learning develop in an appropriate sequence, at a compatible tempo, and with sufficient functional integration when educational demands require their joint operation [57,129,155]. This shifts the explanatory focus from isolated academic deficits toward the developmental organization of learning.

### 7.1. From Component Deficits to Developmental Integration

Reading, attention, and mathematics are not unitary capacities. They are late-emerging cultural, cognitive, and regulatory achievements constructed through the progressive coordination of multiple developing systems [11]. Dyslexia, ADHD, and dyscalculia differ in their predominant manifestations, but each may involve developmental asynchrony among component processes that must become functionally integrated over time [1].

The academic label identifies where difficulty becomes most visible, but not necessarily how that difficulty emerged [2]. Dyslexia is classified primarily through reading difficulty, ADHD through attentional and behavioral dysregulation, and dyscalculia through mathematical difficulty. A developmental timing account instead directs attention toward the processes supporting those outcomes: language, perception, attention, motor control, working memory, executive regulation, symbolic learning, processing speed, and automatization.

The model does not simply add developmental timing as another risk factor. It proposes that the timing and quality of functional integration among systems may constitute an additional source of developmental variation relevant to learning outcomes [10]. Learning difficulties may arise not only when individual component skills are weak, but also when otherwise adequate abilities are developmentally out of phase.

A child may possess a reasonable oral vocabulary but unstable phonological segmentation, adequate visual recognition but inefficient print-sound binding, strong conceptual curiosity but poor temporal regulation of attention, or intuitive quantity sensitivity but weak number-symbol integration [24,121,148]. Such profiles are not well characterized as either global delay or impairment within a single isolated domain [1]. Within this framework, the difficulty is proposed to lie in insufficient coordination among systems that schooling requires children to use together.

Developmental asynchrony should not be equated with uniform immaturity. Children may show age-appropriate or advanced functioning in some areas alongside less stable development in others. What matters educationally is whether the relevant capacities are sufficiently integrated to support fluent performance under the demands of formal instruction.

### 7.2. Developmental Fit and School-Readiness Mismatch

A timing account also reframes the significance of educational transitions. School entry, formal reading instruction, the shift from counting to arithmetic fact retrieval, movement from concrete quantity to symbolic notation, and increasing expectations for independent work all introduce new demands for cross-system coordination [151].

A child may appear to develop a learning disability at one of these transitions, although the underlying vulnerability may have been present earlier. The difficulty becomes visible because schooling requires systems that previously functioned relatively independently to operate rapidly, repeatedly, and in coordination [11,139]. Schooling does not necessarily create the vulnerability, but it may reveal, amplify, or buffer it depending on how closely instructional expectations correspond to developmental readiness [44,57,111].

This places school-readiness mismatch near the center of learning disability theory. Chronological age is an imperfect indicator of developmental coordination because children begin school with heterogeneous profiles of cognitive, linguistic, perceptual, motor, executive, and social-emotional readiness [10,46,55,68,100]. Nevertheless, educational systems generally assume that children of similar ages are comparably prepared to read, write, calculate, sustain attention, inhibit responses, follow multistep sequences, regulate movement, and work toward delayed goals.

These expectations depend upon systems that develop unevenly across children and remain sensitive to developmental context [11,141]. Learning may become vulnerable when institutional timing and developmental timing fall out of alignment [139].

The concept of developmental fit shifts the locus of explanation. Within this framework, difficulty is proposed to emerge through the relationship between the child’s developmental organization and the timing, complexity, and pace of instructional demands. It emerges through the relation between the child’s developmental organization and the timing, complexity, and pace of instructional demands. The same neurodevelopmental profile may consequently produce different outcomes under different educational conditions.

### 7.3. Comorbidity and Shared Developmental Architecture

A second contribution concerns overlap among learning disabilities. Dyslexia, ADHD, and dyscalculia are usually treated as distinct diagnostic entities that sometimes co-occur, yet their frequent overlap challenges sharply bounded interpretations [1,2].

The developmental timing account supports a stronger interpretation: overlap is expected because the disorders depend partly upon shared developmental systems. Processing speed, working memory, temporal sequencing, attentional control, language, motor planning, executive regulation, and symbolic learning contribute across more than one academic domain [3]. When these systems develop unevenly or fail to become efficiently coordinated, the resulting learning profile may not conform to diagnostic boundaries [1].

Comorbidity may reflect shared developmental vulnerabilities rather than the coincidental addition of independent disorders [69,87,127]. This does not imply that dyslexia, ADHD, and dyscalculia are interchangeable. Each has characteristic developmental pathways and predominant educational manifestations. However, their boundaries become more permeable when viewed at the level of the systems upon which academic learning depends.

This position extends both multiple-deficit and transdiagnostic accounts. Multiple-deficit models propose that learning disabilities arise from combinations of probabilistic risk and protective factors rather than single necessary causes [112]. Transdiagnostic approaches emphasize that diagnostic categories do not map neatly onto underlying cognitive or neural mechanisms [1]. The developmental timing account adds that the order, tempo, and coordination of these mechanisms also matter.

The same component weakness may have different consequences depending on when it becomes educationally relevant, which compensatory systems are available, and what demands are imposed at that stage of development [18,34,90,91,149]. Mixed learning profiles should be understood not only as additive combinations of deficits but as dynamic outcomes of shared developmental architecture.

### 7.4. From Developmental Asynchrony to Cascading Academic Consequences

A third contribution concerns developmental cascades. Early asynchrony may alter the child’s subsequent learning environment by reducing successful practice, changing the quality of feedback, slowing automatization, lowering confidence, increasing avoidance, and influencing expectations from teachers, parents, and peers [18,34,90,91,149].

Over time, an initially circumscribed mismatch may broaden into more extensive academic difficulty as reduced practice, compensatory effort, altered motivation, and emotional responses accumulate [62]. The profile observed at assessment may no longer directly reveal the original developmental source. It may instead reflect a combination of primary vulnerability and secondary consequences arising from altered opportunity, inefficient compensation, academic anxiety, avoidance, and reduced self-efficacy [6,33,119].

In dyslexia, early instability in phonological awareness, auditory discrimination, rapid naming, or language–print integration may reduce successful decoding practice, thereby weakening orthographic learning and reading fluency [11,26,58,71,93,96]. In ADHD, altered regulation of attention, reward, arousal, inhibition, and motor timing may reduce effective exposure to instruction and interfere with consolidation [69,146]. In dyscalculia, weak coordination among quantity, number words, Arabic numerals, spatial representations, working memory, and procedures may prolong reliance on effortful counting strategies and delay arithmetic fluency [24].

In each case, the later academic difficulty is not simply the direct expression of an initial deficit. It is partly the cumulative result of transactions among developmental vulnerability, educational opportunity, practice, feedback, compensation, and emotional response.

### 7.5. Testable Predictions

For the developmental timing account to provide more than a descriptive metaphor, it must generate predictions that distinguish it from conventional component-deficit models.

#### 7.5.1. Cross-System Coordination Should Improve Prediction

The model predicts that early cross-system asynchrony will predict later learning disability more accurately than weakness in any single component skill.

Children whose phonological awareness, rapid naming, visual attention, and print experience fail to become sufficiently coordinated should be at greater risk for dyslexia than children showing mild weakness in only one of these systems [56]. Similarly, children whose time perception, reward sensitivity, inhibition, working memory, and state regulation remain poorly coordinated should show greater ADHD-related academic impairment than children with isolated attentional symptoms [16,63,66,148].

The critical predictive variable may be the relation among systems rather than the absolute level of performance within any one system.

#### 7.5.2. Consequences Should Depend on Developmental Timing

The educational consequences of a component weakness should depend on when it occurs relative to instruction. Phonological weakness should be especially consequential when formal reading instruction begins, and children must coordinate phonological awareness with grapheme–phoneme mapping and decoding [121]. Weak number-symbol integration should become most visible when mathematics shifts from concrete quantity toward symbolic notation and formal procedures [7,24]. Executive-regulatory asynchrony should become increasingly impairing as classroom demands require sustained independent work, delayed reward, multistep planning, and internalized self-regulation [16,139,148,151]. The same underlying vulnerability may produce markedly different educational consequences depending on when it intersects with instruction.

#### 7.5.3. Mixed Profiles Should Involve Shared Systems

The model predicts that comorbidity will be associated with greater vulnerability in systems shared across academic domains. Children with overlapping dyslexia and ADHD should show greater difficulty in processing speed, working memory, temporal sequencing, attentional control, or automatization than children with more circumscribed reading difficulty [69,87,127]. Children with co-occurring reading and mathematics difficulties should show broader weakness in symbolic learning, verbal sequencing, working memory, or processing speed than children with relatively isolated dyscalculia [1,2,3].

Mixed profiles should be explained not only by additive deficits but by developmental vulnerabilities that influence multiple systems over time.

#### 7.5.4. Intervention Should Depend on Coordination and Developmental Readiness

The model predicts that intervention effects will depend on developmental timing and the coordination of interacting systems. Intervention should be most effective when it strengthens relevant developmental relations before secondary cascade effects become entrenched [11,44].

Dyslexia intervention should target phonological awareness and decoding while also supporting their integration with print exposure, rapid naming, orthographic learning, attention, and reading fluency [13,22,26,41,96,133]. ADHD intervention should address symptoms while modifying the temporal structure of learning through immediate feedback, shorter goal intervals, external organization, structured pacing, and the gradual internalization of self-regulation [146]. Dyscalculia intervention should strengthen connections among quantity, number words, numerals, spatial representations, working memory, and arithmetic operations rather than teaching procedures in isolation [40,44,97,111].

Successful intervention would be defined not only by improvement in component skills but by increased functional coordination among the systems supporting academic learning.

#### 7.5.5. Educational Context Should Moderate Risk

The model also predicts that educational context will moderate the expression of developmental vulnerability. Orthographic transparency should influence how language–print asynchrony is expressed because the consistency and complexity of grapheme–phoneme mappings affect the form of reading difficulty across writing systems [21,156]. Classroom structure, feedback timing, reinforcement, and task duration should influence ADHD-related academic performance because attentional and regulatory functioning varies with motivation, time-on-task, and state-regulation demands [62,148,149,154]. The pace at which mathematics instruction moves from concrete quantity to symbolic procedure should influence the emergence of dyscalculia because mathematical learning depends upon connections among non-symbolic quantity, number words, written numerals, and procedures [7,24].

Developmental timing is not solely a characteristic of the child. Within the proposed framework, it is conceptualized as relational, reflecting the interaction between the child’s developmental organization and the temporal structure of instruction.

#### 7.5.6. Developmental Trajectories Should Be More Informative than Static Classifications

The theory predicts that longitudinal developmental trajectories will explain educational outcomes more accurately than single cross-sectional assessments. Children with similar levels of performance at one time point may follow markedly different developmental paths depending on the coordination and rate of maturation of the underlying systems. Consequently, developmental trajectories should provide stronger prediction of later educational outcomes than static diagnostic classifications alone.

### 7.6. Implications for Research and Classification

Testing the model will require movement beyond single-time-point measurement of isolated component skills. Longitudinal studies should examine the sequencing, rate, and coordination of developing systems in relation to educational experience [10].

The relevant question is not only whether a child has weak phonology, inhibition, or number sense, but how these abilities develop in relation to one another and to instruction [3,70,112,144]. Growth-curve modelling, cross-lagged designs, intensive longitudinal assessment, person-centered developmental profiles, and classroom-based measures of practice and feedback may determine whether cross-system coordination predicts academic outcomes more accurately than isolated component scores [46,57].

In dyslexia, research should examine developmental relations among phonological awareness, rapid naming, auditory discrimination, visual attention, print exposure, decoding, and fluency [56]. In ADHD, studies should examine relations among time perception, delay tolerance, arousal regulation, motor timing, working memory, response inhibition, and task persistence [16,62,63,66,148]. In dyscalculia, studies should examine relations among quantity, number words, Arabic numerals, spatial number representation, working memory, and procedural fluency [24].

The framework also has implications for classification. A timing-based approach would not eliminate dyslexia, ADHD, or dyscalculia as diagnostic categories. It would supplement them with developmental profiles indicating which systems are vulnerable, how they interact, and when those vulnerabilities become educationally consequential [1,2].

Two children diagnosed with dyslexia may both struggle with reading, but one may show a predominantly phonological–print difficulty, whereas another may display broader vulnerabilities involving rapid naming, attention, and fluency [56,121]. Two children with ADHD may show similar classroom behavior, but one profile may be dominated by reward-timing difficulty and another by state-regulatory instability [63,140,145,148]. Two children with dyscalculia may both struggle with arithmetic while differing in whether their primary vulnerability concerns quantity-symbol integration, spatial representation, working memory, or procedural learning [23,24].

Such profiles would retain the practical value of diagnosis while more adequately representing developmental heterogeneity.

### 7.7. Limitations and Boundaries of the Framework

Developmental timing cannot explain all learning disabilities. Genetic liability, neural organization, language exposure, socioeconomic conditions, instructional quality, emotional factors, sensory functioning, and broader developmental conditions all contribute to educational outcomes [1,10,11].

Nor should developmental asynchrony imply that children will simply grow out of their difficulties. Some developmental differences persist, some generate cascading effects, and others become increasingly entrenched through repeated academic difficulty, reduced opportunity, and altered emotional responses [2]. The value of the framework is not that it replaces genetic, neural, cognitive, educational, or contextual explanations, but that it provides a developmental architecture through which their interactions can be understood.

A further limitation is the need for precise operationalization. Without measurable definitions, developmental timing risks becoming a broad metaphor rather than a testable construct. Studies must specify which systems are being examined, how developmental coordination and asynchrony are quantified, which developmental period is relevant, and how the timing and intensity of educational demands are measured [57,155].

The strength of the theory will depend upon its translation into longitudinal, mechanistic, and intervention research capable of testing developmental sequences, cross-system relations, individual trajectories, and context-dependent outcomes [10,11].

### 7.8. General Synthesis

The principal value of the developmental timing account is both integrative and generative. It proposes that dyslexia, ADHD, and dyscalculia to be understood as distinct but related expressions of developmental asynchrony, while bringing developmental systems theory, multiple-deficit models, and transdiagnostic accounts into a common explanatory structure [1,10].

The framework provides hypotheses concerning why learning disabilities become visible when they do, why they frequently overlap, why initially circumscribed vulnerabilities may broaden through developmental cascades, and why intervention effectiveness may depend partly on developmental readiness rather than chronological age alone [2]. Most importantly, it redirects learning disability theory toward a dynamic developmental question: not simply what is impaired, but whether the systems required for learning have become sufficiently coordinated to meet the educational demands placed upon them.

Learning disabilities may be understood as disorders of developmental fit. Within the proposed framework, difficulty is conceptualized as lying neither entirely within the child nor entirely within the educational environment but partly in the relation between maturational organization and educational timing [10,151]. The framework predicts that academic learning may become vulnerable when schooling requires integrated performance before the relevant systems have stabilized and become available for fluent, coordinated use.

The central contribution of the developmental timing account is thus to propose that learning disabilities may be shaped not only by the integrity of individual cognitive systems but also by the developmental timing through which those systems become functionally integrated and aligned with the changing demands of formal education. Within the present framework, developmental asynchrony and developmental cascades therefore refer to related but distinct processes: asynchrony concerns the relative timing and coordination of interacting systems, whereas cascades describe the downstream consequences that may accumulate as an initial developmental mismatch alters subsequent learning opportunities, practice, and experience.

## 8. Educational and Clinical Implications

The developmental timing framework has potential implications for assessment, identification, educational planning, and intervention. Its central practical proposition is that learning disabilities should be evaluated not only against chronological-age expectations but also against the child’s developmental readiness and the functional coordination of the systems supporting learning [151]. A difficulty in reading, attention, or mathematics may reflect not only weakness in a specific academic skill but also a mismatch between educational demand and the current developmental organization of underlying cognitive, linguistic, perceptual, motor, and executive systems [10,139].

Assessment should ask not only whether a child is performing below expectation, but also which component processes are developing slowly, unevenly, unstably, or in insufficient coordination with one another [2]. This requires a shift from exclusively outcome-based assessment toward a developmental interpretation of how the observed difficulty emerged.

### 8.1. Developmentally Informed Assessment

A developmental timing account favors longitudinal assessment over one-time classification. A single evaluation may identify underachievement, but it may not distinguish between developmental delay, maturational asynchrony, inefficient compensation, incomplete automatization, or the early effects of a developmental cascade [57,129,155]. Longitudinal observation is especially important because developmental vulnerabilities may change form as academic and regulatory demands increase [57,129,155].

A child who initially shows difficulty with rhythm, sequencing, phonological awareness, symbolic mapping, motor planning, response inhibition, or attentional regulation may later present with reading failure, arithmetic difficulty, poor written production, reduced classroom participation, or declining academic confidence [1,57,87]. Assessment should consider developmental trajectories rather than interpreting the child’s current performance as a fixed state. Both domain-specific and cross-domain measures are required [3,12,104,127]. For dyslexia, assessment should include phonological awareness, rapid automatized naming, auditory discrimination and temporal processing, oral language, orthographic learning, decoding, reading fluency, and the efficiency of language–print integration [11,22,26,27,59,71,93,96]. For ADHD, assessment should include inhibition, working memory and executive function, delay tolerance, time perception, state regulation, task initiation and persistence, response variability, and sustained performance under differing feedback and reinforcement conditions [5,14,17,32,64,89,98,109,126,128,135,141,144,146,153]. For dyscalculia, assessment should include non-symbolic quantity processing, symbolic number comparison and mapping, number-word and numerical-symbol knowledge, number-line estimation, place-value and multidigit numerical representation, arithmetic strategy use, working memory, and procedural aspects of calculation [16,33,38,40,47,49,54,97,106,107,110,116,117,121,129,140,147].

The purpose is not merely to identify impairment. It is to characterize the developmental rate, stability, sequencing, and coordination of the systems upon which academic learning depends.

### 8.2. Early Identification Without Premature Determinism

Potential indicators of developmental risk may include difficulty with rhythm, temporal sequencing, automatization, phonological timing, sustained attention, response inhibition, working-memory organization, number-symbol integration, task pacing, or consolidation of newly taught skills [62,63,150].

These signs should not be treated as deterministic predictors of later disability. They should instead be interpreted as evidence that a child may benefit from developmentally calibrated support before formal academic demands intensify [45,50,151]. The value of early identification lies not in predicting inevitable failure but in reducing the likelihood that early developmental asynchrony will broaden through reduced practice, repeated frustration, avoidance, and declining self-efficacy [18,34,90,149].

A developmental timing framework challenges both “wait to fail” approaches and premature diagnostic labeling. Delaying support until a child falls substantially below age expectations allows secondary cascade effects to accumulate [18,34,90,124]. Conversely, assigning a fixed diagnostic interpretation too early on the basis of age-based underperformance alone may mistake temporary developmental unevenness for a persistent learning disability [19,29,45,70].

The preferred approach is early but cautious developmental interpretation: identify which systems are not yet sufficiently coordinated, provide support for their integration, and monitor how the child responds as educational demands evolve [2,139].

### 8.3. Developmentally Calibrated Intervention

The framework proposes that intervention timing is critical. Some children may require preparatory developmental support before, or alongside, direct academic instruction [151]. Intervention should be directed not only toward the visible academic outcome but also toward the systems whose incomplete coordination constrains learning.

For dyslexia, this may involve strengthening oral language, phonological awareness, auditory discrimination, rhythm, articulation, rapid naming, and print-sound integration [121]. Direct reading instruction remains essential, but it may be more effective when the linguistic, attentional, and temporal processes supporting decoding and fluency are addressed concurrently.

For ADHD, intervention may need to support state regulation, task pacing, movement, immediate feedback, external structure, shorter reward intervals, and the gradual internalization of temporal and behavioral control [153,154]. Symptom reduction alone may be insufficient if the child continues to experience difficulty initiating tasks, sustaining effort, organizing time, or consolidating academic learning.

For dyscalculia, instruction may need to coordinate concrete quantity, number words, Arabic numerals, spatial number representations, place value, working memory, and arithmetic procedures before fluent symbolic calculation is expected [4]. Procedural teaching is likely to be most effective when it rests on stable conceptual and symbolic foundations.

More broadly, intervention should support the coordination of interacting systems. Oral language should be linked with print, movement and rhythm with attention and sequencing, quantity with symbol, and spatial representation with number. Repeated successful practice should then support consolidation and automatization [4,121,150].

Such intervention is not merely remedial. It is developmentally preparatory. Its purpose is to help component systems become sufficiently coordinated before repeated academic failure becomes entrenched [151]. The framework proposes that academic fluency may not simply be the product of repetition; rather, the benefits of practice may depend partly on whether the relevant developmental systems are sufficiently organized to profit from that experience, consistent with evidence that the effects of experience and intervention can vary with developmental timing and periods of heightened plasticity [29,45,151,154].

### 8.4. Implications for Classroom Practice

Teachers can reduce developmental mismatch by adjusting the temporal and organizational structure of instruction. Useful strategies may include explicit sequencing, multimodal teaching, immediate feedback, structured transitions, shorter work intervals, movement opportunities, cumulative review, distributed practice, and the gradual fading of external supports [151,153,154].

Reading instruction can be paced to promote stable phonological, orthographic, and semantic integration rather than emphasizing speed before accuracy and automaticity have developed [121]. Mathematics instruction can move deliberately from concrete quantity to verbal and spatial representation, written notation, and procedural fluency [4]. Support for attention and self-regulation can externalize time through visual schedules, timers, reminders, proximal goals, predictable routines, and structured feedback [154].

These strategies do not lower academic expectations. They alter the route and timing through which children are supported in reaching them. Their purpose is to increase the likelihood that instruction is delivered in a form and at a pace that the child’s developing systems can use effectively [151].

A developmental timing perspective also suggests that responsiveness to intervention should itself become part of assessment. Improvement following structured developmental support may indicate that the principal difficulty lies in insufficient coordination, limited practice, or a mismatch between instructional format and developmental readiness. Limited response may indicate the need for more intensive, specialized, or differently targeted intervention.

### 8.5. Interdisciplinary and Family-Centered Support

The framework further encourages collaboration among educators, psychologists, speech-language specialists, occupational therapists, physicians, and families. Learning disabilities rarely involve a single academic process in isolation, and intervention is unlikely to be optimal when professional disciplines address separate symptoms without coordinating their objectives.

A child who struggles with reading may also require support for oral language, attention, rhythm, motor planning, working memory, or emotional regulation. A child with ADHD may need academic instruction structured around immediate feedback, manageable time intervals, movement, and repeated opportunities for consolidation. A child with dyscalculia may require coordinated spatial, verbal, symbolic, and procedural supports within mathematics teaching.

Families also provide essential information about developmental history, changing patterns of difficulty, response to instruction, motivation, and functioning across settings. Their participation can help determine whether the difficulty is stable, context-dependent, worsening, or responsive to changes in pacing and support.

Effective intervention should be coordinated across systems and settings, consistent with the framework’s proposition that learning difficulties may reflect interactions among developmental systems. The practical objective is not simply to remediate a weak academic skill but to create conditions in which the relevant systems can become increasingly stable, integrated, and available for successful learning.

## 9. Conclusions

Learning disabilities are not only disorders of reading, attention, or mathematics. They may also reflect developmental asynchrony among the neural, cognitive, linguistic, perceptual, motor, and executive systems upon which academic learning depends. Dyslexia, ADHD, and dyscalculia differ in their predominant expression but involve multiple, partly overlapping component processes [3,104,112,127]. The developmental timing framework proposes that successful academic functioning depends not only on the integrity of these component processes but also on their progressive functional integration over development.

Reading depends on the coordination of oral language, phonological processing, print recognition, attention, working memory, orthographic learning, and fluency [121]. Successful attention and classroom regulation depend on interactions among arousal, inhibition, motivation, working memory, motor control, temporal organization, and future-directed action [16,139,148]. Mathematical learning depends on the integration of quantity, symbol, space, language, working memory, and procedural sequencing [24].

The developmental timing account proposes hypotheses concerning why learning disabilities often become visible during educational transitions, why they frequently overlap, how initially circumscribed developmental differences may broaden through cascade processes, and whether intervention effectiveness may depend partly on developmental readiness rather than chronological age alone [1,2]. It does not diminish the value of domain-specific diagnoses. Instead, it places them within a broader account of developmental coordination, educational demand, heterogeneity, and change over time [10,11].

The central claim is not that timing constitutes the sole cause of learning disabilities. Genetic, neural, cognitive, experiential, instructional, and contextual factors remain essential. The contribution of the framework is to identify developmental timing and functional integration as dimensions that are underemphasized when learning disabilities are classified primarily by their academic outcomes.

Children may struggle not only because an individual component skill is weak, but because the systems required for successful learning have not become sufficiently coordinated when formal education requires their joint operation. The framework proposes that learning difficulties emerge partly through interactions between the child’s developmental organization and the temporal structure of educational demand.

This account supports earlier identification, more precise developmental interpretation, longitudinal assessment, and intervention directed toward strengthening relations among the systems supporting learning. Its central practical implication is not that academic skills should be postponed until all component processes are fully mature, but that instruction should be calibrated, scaffolded, and sequenced so that children can progressively integrate those processes through successful learning experiences.

Learning disabilities may thus be understood as disorders of developmental fit. The framework predicts that learning difficulties may become most likely when the timing of neural and cognitive integration falls out of alignment with the pace, sequence, and complexity of formal education. From this perspective, improving developmental fit may require more than remediating isolated skills; it may also require supporting the functional coordination of the developmental systems involved in fluent academic learning. Future research should determine whether developmental timing can be operationalized into reliable longitudinal markers capable of identifying children at risk before substantial educational divergence occurs. If supported empirically, a developmental timing framework could complement existing diagnostic approaches by shifting emphasis from the identification of established learning difficulties toward the prediction, prevention, and developmental optimization of learning trajectories.

## Figures and Tables

**Table 1:** Summary of the principal developmental timing vulnerabilities proposed across dyslexia, ADHD, dyscalculia, and mixed learning profiles.

Profile	Surface Academic Expression	Component Systems Involved	Proposed Developmental Asynchrony	Likely Cascade Pathway	Intervention Implication
Dyslexia	Difficulty with decoding, spelling, word recognition, reading fluency, and later comprehension	Phonological awareness, auditory temporal processing, speech-sound discrimination, visual-symbol recognition, rapid automatized naming, orthographic learning, working memory, attention, articulation	Language, phonology, print, attention, and fluency systems are not sufficiently synchronized when formal reading instruction begins.	Slow or inaccurate decoding reduces successful print exposure, weakens orthographic learning, delays fluency, increases effort, and may reduce reading practice and confidence.	Strengthen phonological awareness, auditory discrimination, rhythm, rapid naming, print-sound mapping, orthographic learning, and fluency through structured, developmentally paced instruction.
ADHD	Difficulty with attention, task initiation, inhibition, persistence, classroom regulation, completion of work, and academic consolidation	Arousal regulation, inhibition, reward timing, working memory, time perception, task pacing, motor timing, executive control, state regulation	Executive, motivational, attentional, motor, and state-regulatory systems are not aligned with the temporal demands of classroom learning.	Missed instructions, delayed task initiation, incomplete practice, weak consolidation, inconsistent performance, negative feedback, and reduced academic self-efficacy	Externalize time, provide immediate feedback, shorten goal intervals, structure transitions, allow movement, support task pacing, and gradually build internal regulation
Dyscalculia	Difficulty with number facts, calculation, place value, numerical comparison, arithmetic fluency, and mathematical reasoning	Approximate magnitude, exact quantity, number words, Arabic numerals, spatial number lines, place value, working memory, executive sequencing, procedural learning	Quantity, language, spatial, symbolic, memory, and procedural systems are not sufficiently synchronized when symbolic mathematics is introduced.	Weak number-symbol mapping increases counting dependence, delays fact retrieval, raises working-memory load, slows procedural fluency, and contributes to avoidance or math anxiety.	Link concrete quantity, number words, Arabic numerals, spatial number lines, place value, and arithmetic procedures before expecting fluent symbolic calculation
Mixed learning profiles	Cross-domain difficulties involving reading, attention, writing, mathematics, processing speed, working memory, or classroom regulation	Processing speed, working memory, temporal sequencing, language, attention control, motor planning, symbolic mapping, executive regulation	Shared timing-sensitive systems affect more than one academic domain, producing overlap across diagnostic categories	Vulnerability in one system affects multiple learning contexts, leading to comorbidity, shifting symptom profiles, cumulative academic difficulty, and inefficient compensation	Assess shared component systems rather than only domain-specific achievement; design interventions that coordinate language, attention, memory, motor timing, sequencing, and symbolic learning

## Data Availability

No new data were created or analyzed in this study. Data sharing is not applicable to this article.
